# The role of hydrogen sulfide in aging and age-related pathologies

**DOI:** 10.18632/aging.101026

**Published:** 2016-09-27

**Authors:** Bernard W. Perridon, Henri G.D. Leuvenink, Jan-Luuk Hillebrands, Harry van Goor, Eelke M. Bos

**Affiliations:** ^1^ Department of Pathology and Medical Biology, University Medical Center Groningen, the Netherlands; ^2^ Department of Surgery, University Medical Center Groningen, the Netherlands; ^3^ Department of Neurosurgery, Erasmus Medical Center Rotterdam, the Netherlands

**Keywords:** hydrogen sulfide, H_2_S, aging, hallmarks of aging, gasotransmitters

## Abstract

When humans grow older, they experience inevitable and progressive loss of physiological function, ultimately leading to death. Research on aging largely focuses on the identification of mechanisms involved in the aging process. Several proposed aging theories were recently combined as the ‘hallmarks of aging’. These hallmarks describe (patho-)physiological processes that together, when disrupted, determine the aging phenotype. Sustaining evidence shows a potential role for hydrogen sulfide (H_2_S) in the regulation of aging.

Nowadays, H_2_S is acknowledged as an endogenously produced signaling molecule with various (patho-) physiological effects. H_2_S is involved in several diseases including pathologies related to aging. In this review, the known, assumed and hypothetical effects of hydrogen sulfide on the aging process will be discussed by reviewing its actions on the hallmarks of aging and on several age-related pathologies.

## INTRODUCTION

Over the past decades the worldwide human life expectancy has increased substantially [1]. This, combined with several other factors, led to a dramatic and ongoing increase in the proportion of the world's human population aged over 60 years [2]. As a consequence, the number of people experiencing age-related deterioration in health is rising, which will cause a great burden on the healthcare system [2]. The deterioration of the physiological integrity leads to an increased incidence of major human pathologies and increases the probability of death [1, 3]. Age remains the main risk factor for the development of debilitating and life-threatening, age-related pathologies such as cardiovascular disease, cancer, type 2 diabetes mellitus and neurodegenerative disorders including dementia [1–4]. Understanding what causes aging and how aging and age-related diseases are interrelated is therefore essential. This led to an increased interest in aging research and made healthy aging a hot topic in research.

Research on the aging process of sometimes distantly related model organisms – including yeast, nematodes, flies, mice and humans – led to the idea that lifespan regulation and aging are modulated by common and conserved mechanisms in many, if not all species [3, 5]. The discovery that exogenous hydrogen sulfide (H_2_S) prolongs the lifespan of the nematode *Caenorhabditis elegans* [6] has led to new insights in its role in health and disease, suggesting a relationship between H_2_S and aging [7]. This gaseous molecule was not considered to be physiologically relevant until the 1990s. Since then, increasing evidence showed its beneficial effects in several disease models, including age-related pathologies [8]. The age-dependent decline in plasma H_2_S levels found in human subjects 50 to 80 years of age supports the link between H_2_S and aging [9]. However, the precise relationship between H_2_S and aging is still largely unknown as both the role of H_2_S in the aging process as well as the effects of aging on the metabolism of H_2_S are not fully understood yet [8, 10]. This review outlines the current knowledge on the relationship between hydrogen sulfide and aging so as to determine the potential beneficial effects of hydrogen sulfide in aging and age-related pathologies.

## THE AGING PROCESS

Aging is the progressive loss of physiological function which emerges when organisms grow older, with death of the organism as the ultimate, inevitable consequence [1, 3, 11]. Classic symptoms of human aging include graying and loss of hair, loss of hearing and eyesight, reduced fertility, immune system failure and loss of cognition [2]. These classical symptoms decrease the quality of life of most elderly, but are generally accepted as inevitable consequences of aging. These symptoms are believed to result from time-dependent accumulations of cellular damage leading to a gradual loss of function at the molecular, cellular, tissue and organismal level [12, 13]. A variety of molecular, biochemical and metabolic alterations occurring at the cellular level are thought to cause these functional losses [2]. For many years, aging research was largely focused on identifying the underlying cellular mechanisms as to find novel drug targets to modulate the aging process and to attain healthy aging by delaying the onset of age-related pathologies [3]. This approach has led to some unsubstantiated and opposing claims for potential cures as opposed to disease-oriented research [14]. It turned out that the aging processes are highly complex with numerous mechanisms and pathways involved. Several theories and mechanisms explaining the biology of aging have been proposed [2]. These theories are not mutually exclusive and attempts to unify them into one theory have not yet succeeded. Therefore, the molecular and cellular pathways generally considered to contribute to the process of aging were categorized and proposed as the ‘hallmarks of aging’ [15].

### Hallmarks of aging

**Figure 1:**
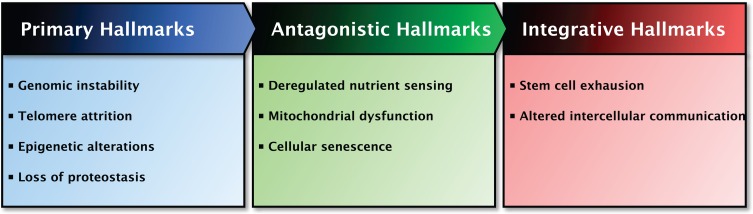
Overview of the Hallmarks of Aging and their functional interactions The proposed nine hallmarks of aging are categorized based on common characteristics and their contribution to aging. Left panel: The primary hallmarks of aging are the hallmarks regarded as the primary cause of cellular damage. Middle panel: The antagonistic hallmarks of aging are those hallmarks considered to be part of compensatory or antagonistic responses to damage. These hallmarks initially mitigate the damage, but eventually can become deleterious themselves. Right panel: The integrative hallmarks of aging are the end result of the two previously described categories and are ultimately responsible for the functional decline associated with aging. The interactions between the categories are indicated at the top of the panels.

In total, nine hallmarks of aging were proposed which together are thought to determine the aging phenotype (Figure 1) [15]. The criteria for the hallmarks are that each hallmark should be manifested during normal aging and that its experimental intervention should both accelerate or retard the normal aging process, depending on the intervention [15]. However, not all hallmarks currently meet up to all the criteria as amelioration of the aging process is not always successful [15]. The hallmarks are interconnected, making it difficult to determine the relative contribution of each hallmark to aging. There is some degree of hierarchy between the hallmarks of aging and therefore they are divided into three categories: primary, antagonistic and integrative hallmarks [15]. Primary hallmarks, including *genomic instability*, *telomere attrition*, *epigenetic alterations* and *loss of proteostasis*, are considered to be the primary cause of damage at cellular level which progressively accumulates with time. In response, antagonistic hallmarks that are principally beneficial and mitigate damage may become deleterious themselves, progressively contributing to aging. *Deregulated nutrient sensing*, *mitochondrial dysfunction* and *cellular senescence* belong to this category. Finally, the integrative hallmarks arise when the tissue homeostatic mechanisms cannot compensate for the damage caused by the previous two categories. The integrative hall-marks, *stem cell exhaustion* and *altered intercellular communication*, are ultimately responsible for the age-related functional declines [15]. All nine hallmarks of aging and the effects of H_2_S on each hallmark will be comprehensively discussed in this review.

## GASOTRANSMITTERS

As research on aging is as old as the hills, it has witnessed the emergence of several new fields of research. Among them is the investigation on gasotransmitters: small, labile and endogenously-generated gaseous transmitters that mediate physiology and disease. Nitric oxide (NO) and carbon monoxide (CO) were the first two identified gasotransmitters and based on their biology several criteria to classify gasotransmitters were proposed [16]. Gasotransmitters diffuse freely across membranes and have well-defined functions in signal transduction, acting on specific cellular and molecular targets at physiologically relevant concentrations. Their endogenous production is regulated by specific substrates and enzymes in mammalian cells and their function can be mimicked by exogenously applied counterparts or obtained from the diet [16].

Nowadays, hydrogen sulfide (H_2_S) is acknowledged as the third gasotransmitter [8, 16]. The biological and medical importance of the gasotransmitters NO, CO and H_2_S is now widely recognized. Whereas the endogenous concentrations of each gasotransmitter in the circulation or in tissues are relatively low, these concentrations are sufficient to execute their specific physiological actions [17–19]. Recently, other small gaseous molecules were evaluated for their candidacies as gasotransmitter and as ammonia (NH_3_) did meet all criteria for gasotransmitters it should be classified as the fourth gasotransmitter [17]. Over the past decade, evidence demonstrating the importance of gasotransmitters to the human body mounted as their regulatory capacities in controlling important physiological functions, like vascular tone, defense against pathogens, neuro-modulation, apoptosis and energy metabolism, were shown [20].

Despite the fact that all gasotransmitters have their own specific functions and targets, it is plausible that interactions take place between their signaling roles [21]. Indeed, gasotransmitters share several targets and functions with one another [16, 20]. The activities of these shared targets are, however, modulated through different mechanisms [8, 22, 23]. Another possibility is that the eventual outcome of the actions of gasotransmitters is the same despite the fact that different targets and mechanisms were involved [17]. Studies also revealed crosstalk between gaso-transmitters [22–24] in such a way that “each gas may antagonize, reciprocally regulate or potentiate the cellular effects of each other through their production, downstream molecular targets and direct chemical interactions” [21]. Their interconnectedness complicates the search for the specific effects of each individual gasotransmitter, as observed beneficial effects after the modulation of one gasotransmitter might be the result of its crosstalk with other gasotransmitters.

## HYDROGEN SULFIDE

Historically, hydrogen sulfide was known as a toxic gas characterized by the strong odor of rotten eggs. Until the 1990s, the research on H_2_S was mainly focused on its toxicity. Accidents in industrial settings showed the danger of the gas, as exposure to high concentrations of H_2_S caused collapse, unconsciousness and respiratory paralysis, and ultimately led to death [25]. Since then, new knowledge has positively transformed the way H_2_S is perceived, as studies discovered physiological functions of H_2_S in biological systems [26, 27] and showed endogenous production of H_2_S in many parts of the mammalian body [8, 28]. As H_2_S is soluble in both water and lipids, it easily penetrates biological membranes without facilitation of membrane channels [29]. The term ‘H_2_S’ in this review refers to H_2_S and its anions, as more than 80% of this weak acid dissociates to hydrosulfide anion (HS^−^) and proton (H^+^) at the physiological pH (pH=7.4) in the circulation and the cells [30]. The contribution of the other sulfide anion (S^2−^) is negligible at physiological conditions, as the dissociation of HS^−^ to S^2−^ occurs almost exclusively at high pH [29, 30]. It is not possible to separate the effects of H_2_S and HS^−^ on physiological functions and signaling processes since these species coexist at physiological conditions in cells and in circulation [29, 31, 32].

### Endogenous and exogenous sources of H_2_S

#### Endogenous production

Hydrogen sulfide is endogenously produced by most mammalian cells via enzymatic and non-enzymatic pathways [8, 28]. However, the contribution of the non-enzymatic pathway, in which elemental sulfur or organic polysulfides are reduced to H_2_S, is small [16, 33, 34]. Stressful conditions, like increased oxidative stress and hyperglycemia, promote H_2_S production from this pathway [8].

**Figure 2:**
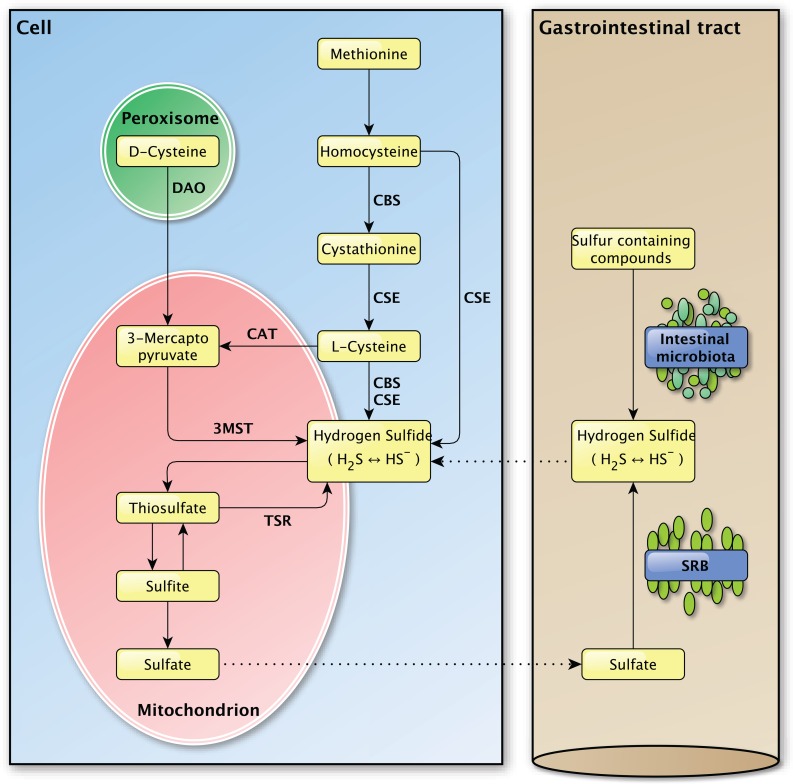
Overview of the endogenous and exogenous H_2_S production in the mammalian body Left panel: The endogenous production of H_2_S in mammalian cells. Several important enzymes are mentioned along the arrows. Right panel: The exogenous production of H_2_S in the gastrointestinal tract by the intestinal microbiota and sulfate-reducing bacteria, for which the H_2_S production is endogenous. The dashed lines between the left and the right panel indicate the transport of molecules between the compartments.

The enzymatic production of H_2_S depends on three enzymes, namely cystathionine γ-lyase (CSE), cystathionine β-synthase (CBS) and 3-mercapto-pyruvate sulfurtransferase (3MST) (Figure 2) [35, 36]. The vitamin B_6_-dependent enzymes CBS and CSE are normally localized in the cytoplasm and translocate to mitochondria under stressful conditions to promote the mitochondrial production of H_2_S and adenosine triphosphate (ATP) [37–39]. The other H_2_S producing enzyme 3MST is mainly localized in mitochondria and is found in the cytoplasm to a minor extent [40, 41].

Sulfur-containing amino acids, like methionine and cysteine, are the main precursors for the enzymatic generation of H_2_S. CBS and CSE account for the majority of the endogenous produced H_2_S in mammalian tissues [16]. Their expression is tissue specific and was identified in liver, kidney and brain cells, skin fibroblasts and blood lymphocytes among others [16].

After the conversion of methionine to homocysteine, CBS is required to form cystathionine from homocysteine whereupon CSE converts cystathionine to L-cysteine, the latter being the key substrate in the generation of H_2_S. Hydrogen sulfide can also be generated directly from homocysteine by CSE, but not by CBS [42]. L-cysteine is used by CBS and CSE to form H_2_S and by cysteine aminotransferase (CAT) to produce 3-mercaptopyruvate, the main substrate for H_2_S production by 3MST. In the presence of D-amino acid oxidase (DAO), 3-mercaptopyruvate can also be produced from D-cysteine in peroxisomes [43]. The presence of a dithiol is required for 3MST in order to release H_2_S [44]. *In vivo*, H_2_S is metabolized by methylation in the cytosol [16] or by oxidation in mitochondria, whereupon it is secreted as sulfite, thiosulfate and sulfate [36]. *In vivo*, hydrogen sulfide can be re-formed from sulfite and thiosulfate, but not sulfate [39, 45].

#### Exogenous production

Aside from endogenous H_2_S production, H_2_S can be produced by bacteria in the mouth and the gastrointestinal tract (Figure 2). From the perspective of the host-organism, this H_2_S production is exogenous as it is produced by bacteria in the external milieu of the host and not by that organism itself. Sulfur-reducing bacteria, for example, are part of the normal intestinal microbiota in healthy individuals and have been identified in human feces [46]. Hydrogen sulfide is synthesized by these bacteria from alimentary as a consequence of their metabolic activity in which they use sulfate as a terminal electron acceptor [8, 47]. The main source of this inorganic sulfate is the aliment, however the secretion of sulfate by the host gastrointestinal tract may also be an important source [48]. As the intestine is highly permeable to H_2_S [49], the production of high concentrations of H_2_S by these bacteria would be expected to cause severe tissue damage [50]. The exogenous production of H_2_S was also suggested to play a role in several pathologies of the intestinal tract, like inflammatory bowel disease and colorectal cancer, but direct links are yet to be established [51–53]. The intestinal mucosa is thought to protect the gastrointestinal tract against high concentrations of H_2_S by a specialized detoxification system which rapidly and effectively metabolizes H_2_S to thiosulfate and sulfate [8, 50], whereupon sulfate can be secreted [48]. It is possible that defects in this detoxification pathway play a part in intestinal pathologies [49, 50]. The importance of proper H_2_S catabolism is demonstrated in patients with ethyl-malonic encephalopathy, a disease in which malfunction of the persulfide dioxygenase ETHE1 results in multi-organ pathology in the brain, gastrointestinal tract and peripheral vessels for example [54]. Due to mutations in the *ETHE1* gene [55], the inorganic sulfur catabolism is impaired which leads to the accumulation of H_2_S and various H_2_S-related toxic effects in different tissues [54, 56, 57]. Decreased exogenous H_2_S production, due to treatment with the antibiotic metronidazole, and improved H_2_S buffering, by administration of the glutathione precursor *N*-acetylcysteine, caused marked clinical improvements in patients with ethylmalonic encephalopathy [58]. The exact role of exogenously produced H_2_S in (patho-) physiology still needs to be determined and separated from the effects of endogenously produced H_2_S [53].

### Exogenous administration

In experimental settings, levels of H_2_S can be manipulated by the administration of exogenous H_2_S or H_2_S releasing compounds. Sulfide-sodium salts, exposure to gaseous H_2_S, slow-releasing H_2_S donors, hybrids of H_2_S-donors and known substances, thiosulfate, cysteine analogs and modulation of the expression or activity of H_2_S producing enzymes are several options for altering H_2_S levels in experimental settings.

## HYDROGEN SULFIDE AND (PATHO-) PHYSIOLOGY

Nowadays, H_2_S is recognized as an important signaling molecule with various (patho-)physiological effects and was shown to be involved in cardiovascular system diseases, cancer and neurodegenerative diseases [8, 10]. The most widely studied functions of endogenous H_2_S relate to its vasodilatory effects [23, 59, 60] and its ability to reduce and modulate oxidative stress. Additionally, several other physiological function of H_2_S signaling were proposed [10]. These studies demonstrated that H_2_S has the potential to provide beneficial effects on health in many (patho-)physio-logical processes and age-related pathologies. However, the effects of H_2_S on aging are not that commonly studied. Nevertheless, pathways of most – if not all – hallmarks of aging are found to be influenced by H_2_S. Studies that identified the effects of H_2_S on these pathways are thus relevant in the light of aging and are described next.

## HYDROGEN SULFIDE AND AGING

### Role of H_2_S in the primary hallmarks of aging

#### Genomic instability

Exogenous and endogenous treats continuously challenge genome integrity and stability [61]. Organisms have evolved a complex network of repair, damage tolerance and checkpoint mechanisms to counteract most damage caused to the nuclear genome [15, 61, 62]. Artificial reinforcements in these mechanisms extended healthy lifespan in mice [63] and were suggested to delay aging [15]. In contrast to the nuclear genome, the protection of mitochondrial DNA is not that efficient [64] and is heavily dependent on the machinery of nuclear DNA repair [65]. Besides that, mitochondrial DNA is more vulnerable to mutations due to the oxidative microenvironment of the mitochondria and the lack of protective histones on mitochondrial DNA [64]. Therefore, aging-associated mutations and deletions in the mitochondrial genome may also contribute to the aging process [66].

Besides the destabilizing effects of alterations in the nuclear and mitochondrial genome, the stability of the genome can also be deteriorated by defects in the nuclear architecture [67]. Alterations in the nuclear lamina have been detected in disease and during normal aging [68, 69], causing changes in several stress pathways [70, 71] and attrition of adult stem cells [72, 73]. The lifespan of progeroid mice was extended after these pathways were restored [71, 74], indicating their importance in the aging process.

##### Effect of H_2_S on genome stability

Genomic stability can be affected by H_2_S, but the data are conflicting. In Chinese hamster ovaries, human colonic epithelial cells and human lung fibroblasts, H_2_S may exert genotoxic actions [75, 76]. Human fibroblasts treated with NaHS showed a concentration-dependent increase in the formation of micronuclei, indicating DNA damage, and were propelled towards cell-cycle arrest and apoptotic death through DNA damage responses involving p53, p21 and the apoptosis regulators, Bax and cytochrome c [76]. H_2_S was genotoxic at concentrations of 0.25 mmol/L, which is similar to the physiological concentration observed in mouse colon (0.2–1 mmol/L [77]) and human feces (0.2–3.4 mmol/L H_2_S [78]) [75]. However, the genotoxic concentration is much higher than the human plasma concentration of H_2_S (10–100 μmol/L) [79, 80]. High, genotoxic H_2_S concentration in the gut, possibly due to production by commensal sulfate-reducing bacteria, may play a role in the genomic instability and the acquisition of mutations observed in colorectal cancer [75]. However, it must be noted that the reliable measurement of H_2_S is still a topic of discussion, so it is not known whether these mentioned plasma concentrations are accurate or comparable between studies [32, 81–85].

Others point out the positive effects of H_2_S on genome stability. Hydrogen sulfide attenuates DNA damage in human endothelial cells and fibroblasts by increasing MEK1 S-sulfhydration, ERK1/2 and PARP-1 activity leading to the activation of DNA damage repair mechanisms and protection from cellular senescence [86]. The CSE/H_2_S pathway is important in genome stability and cell proliferation, as its inhibition in hepatoma cells decreased their proliferation, enhanced ROS production and mitochondrial disruption, pronounced DNA damage and increased apoptosis [87]. Proliferation was decreased due to downregulation of ERK1/2 activity [87]. The increased apoptosis after H_2_S signal inhibition was associated with the activation of p53, p21, Bax and other pro-apoptotic factors [87]. Thus, both high [76] as well as low H_2_S levels [87] can induce apoptosis. Two other studies showed protective effects of H_2_S containing water [88] and a mitochondrially-targeted H_2_S donor (AP39) [89] against oxidative stress and oxidative DNA damage in peripheral blood mononuclear cells of Alzheimer's patients and a murine brain microvascular endothelial cell line, respectively, again showing the positive effects of increased intracellular H_2_S on nuclear and mitochondrial genome stability. These and other studies also described improved cell viability [88, 90] and affected mitochondrial activity [89] after H_2_S administration.

#### Telomere attrition

The genomic stability systems also include specific mechanisms for maintaining the appropriate length and functionality of telomeres [91]. Telomere maintenance is, however, considered as a distinct hallmark of aging. The unique DNA-protein structure of telomeres is essential for maintaining genomic integrity, as they cap the terminal ends of linear chromosomes in order to prevent appearing as DNA double-strand breaks in need of repair and to protect these ends from degradation [92]. Normal aging in mammals is accompanied by the progressive and cumulative loss of telomere length and function as a consequence of normal replication, due to oxidative damage or as a result of replication errors [15, 92, 93]. Telomerase, a specialized DNA polymerase, is required to elongate telomeres as the replicative DNA polymerase lacks this capacity. However, most mammalian somatic cells do not express telomerase, making telomeres particularly susceptible to age-related deteriorations [91]. When telomeres become too short or when too much damage has occurred, they become dysfunctional. Dysfunctional telomeres are highly efficient in inducing apoptosis and/or cellular senescence and accelerate aging in mice and humans [15, 94, 95]. Experimental stimulation of telomerase was shown to delay or even reverse aging [15, 96].

##### Effect of H_2_S on telomere maintenance

No studies have specifically described the effect of H_2_S on telomere maintenance, but the described protective effects of physiological levels of H_2_S on genome stability might also preserve telomeres and prevent telomere attrition and dysfunction.

#### Epigenetic alterations

Along with the genetic components in aging, a role for non-genetic factors in the aging process is implicated [97]. Epigenetic alterations such as posttranslational modification of histones, alterations in DNA methylation patterns and chromatin remodeling [15, 97–99], can regulate the accessibility of the DNA and underlie the differential gene transcription observed between cell types, developmental stages and disease states [100–102]. Several histone modifications are associated with aging [103, 104], such as the deacetylase activity of sirtuins. Manipulation of a single sirtuin gene in several animal models resulted in noteworthy effects on longevity by slowing down organismal aging [105]. At least three members of the sirtuin family, namely SIRT1, SIRT3 and SIRT6, are involved in the regulation of longevity and/or healthy aging in mammals [15]. The underlying mechanisms by which they affect these processes differ between the sirtuins [15].

Besides histone modification, DNA methylation also alters chromatin structure and regulates gene expression [106]. Alterations in DNA methylation are considered to be a central mechanism in normal development, some diseases and in the aging process [107] and accumulate during life, as was shown in a study in centenarians and newborns [108]. However, the relationship between DNA methylation and aging is complex, as both a global decrease in DNA methylation, as well as specific age-related hypermethylation at certain developmentally regulated genes, including various tumor suppressor genes and Polycomb target genes, were described in different human tissues during aging [15, 98, 108, 109]. Among the differentially methylated regions, some were shown to be related with age or were associated with age-related phenotypes [110]. Nevertheless, direct experimental evidence showing that organismal lifespan can be extended by altered DNA methylation patterns was not found thus far.

Several other key chromosomal protein and chromatin remodeling factors are diminished in normal and pathological aged cells [111, 112]. Alterations in their expression altered the lifespan of flies [113], demonstrating their role in the aging process.

##### Effect of H_2_S on epigenetics

Several studies show epigenetic chromatin modulation by H_2_S [114, 115]. Slow-releasing H_2_S donors inhibit tumor growth both *in vitro* and *in vivo* by a combination of cell-cycle arrest and apoptosis promotion, which was related to histone hyperacetylation [116–118]. The transcription of pro-inflammatory cytokines, like IL-6 and TNF-α, was altered due to histone modifications and accompanying changes in chromatin structure [114]. Expression and activity of SIRT1 is mediated by H_2_S [10, 119, 120]. The protective effects of H_2_S against senescence [119, 121], apoptosis [90] and neurotoxicity of formaldehyde [120] were found to be modulated through SIRT1 activity and were attenuated by SIRT1 inhibition [90, 120]. Direct anti-apoptosis and antioxidant effects of H_2_S via the SIRT1 pathway were demonstrated in cells under oxidative stress [90, 122].

H_2_S is also associated with altered DNA methylation. High plasma levels of homocysteine inhibit the CSE/H_2_S signaling and trigger mitochondrial toxicity, endothelial dysfunction and inflammation by increasing DNA methylation and transcriptional alterations, like elevation of TNF-α and IL-1β plasma levels and inhibition of CSE expression [123, 124]. Supply of H_2_S-releasing compounds rescued these cells from the harmful effects of high levels of circulatory homocysteine [123, 124]. Hydrogen sulfide alters the epigenetics of the mitochondrial genome, contributing to the replication of mitochondrial DNA, cellular energy metabolism and mitochondrial bioenergetics [125]. Exogenous H_2_S repressed the expression of DNA methyltransferase in cultured smooth muscle cells and aortic tissues from mice, resulting in the demethylation of certain regions of the mitochondrial genome, whereas CSE deficiencies showed opposite effects [125]. These studies demonstrate that epigenetics can be changed by alterations in the transsulfuration pathway.

#### Loss of proteostasis

Many studies demonstrated the importance of a proper protein homeostasis or ‘proteostasis’ in health and disease. Permanent or temporal alterations in proteostasis, as a result of changes in physiology or exposure to environmental stresses, are a common feature of development and cellular and organismal aging [126, 127]. Several age-related pathologies, like Alzheimer's and Parkinson's disease, are characterized by the appearance of misfolded or aggregated proteins with disease-causing, proteotoxic effects [128, 129]. In order to preserve the integrity, stability and functionality of the proteome, all cells take advantage of a network of quality control pathways that coordinate the synthesis, folding, disaggregation and degradation of proteins and their posttranslational modifications [15, 127]. This proteostasis network includes the translational machinery, molecular chaperones, the ubiquitin-proteasome system and the lysosomal autophagy machinery [129]. Several signaling pathways were shown to assist and modify the proteostasis network but are not considered as direct components of that network [127]. Molecular chaperones, like proteins of the heat-shock family, mediate the folding and stabilization of correctly folded proteins [126, 130]. Studies showed the importance of chaperones in the regulation of lifespan in several model organisms [131–133]. However, the efficacy of protein quality control by these chaperones declines with aging [134]. Affected proteins are normally targeted to destruction by the ubiquitin-proteasome or the lysosomal autophagic pathways [126, 135]. However, the activities of these proteolytic systems also decline with aging [136, 137]. Numerous studies showed that the induction of these pathways extended longevity in several animal models [136, 138–141]. Together, these studies support the idea that collapsing proteostasis contributes to the aging process.

##### Effect of H_2_S on proteostasis

Hydrogen sulfide affects the maintenance of cellular protein homeostasis. NaHS treatment suppresses the increased protein synthesis and aggregation in cultured brain slices from Zucker diabetic rats, normalizing proteostasis and counteracting oxidative stress [142]. NaHS inhibited the formation of advanced glycation end products, which disrupt proteostasis, in human neuroblastoma SH-SY5Y cells exposed to D-galactose [143]. Exposure to H_2_S protected animals against hypoxia-induced disruptions of proteostasis, like protein aggregation and cytotoxicity, and reversed the detrimental effects of hypoxia on protein homeostasis [144]. Cardioprotective effects of heat-shock protein-90 were observed in chemical hypoxia-induced injury to rat H9c2 cardiomyoblasts after NaHS exposure, inhibiting oxidative stress and preserving mitochondrial function [145]. These effects might be a result of improvement to cellular proteostasis. H_2_S may also affect proteins through S-sulfhydration, which is a form of posttranslational modification, leading to changes in intracellular signaling [146].

In the premature aging disorder Werner syndrome, fibroblasts are highly stressed with extensive protein production and aggregation in the cytosol accompanied by nuclear dysmorphia [147]. Treatment with NaHS or rapamycin normalized the morphological phenotype and restored proteostasis by blocking mTOR activity and annulling protein aggregations [147]. These studies demonstrate that H_2_S can influence the maintenance of cellular protein homeostasis.

### Role of H_2_S in the antagonistic hallmarks of aging

#### Deregulated nutrient sensing

Several nutrient signaling pathways have been linked to aging. Restricting the dietary caloric intake is the most effective intervention known to slow down aging and extend lifespan in many species [14, 148]. The insulin/IGF1 signaling pathway is the most conserved glucose sensing and the best-characterized aging-controlling pathway [15]. Attenuations at different levels in this pathway were associated with lifespan extension in animals [149–152] and humans [153, 154]. Among the downstream modulators of the insulin/IGF-1 network, the FOXO transcription factors are the most relevant [149], as they modulate the expression of pro-longevity genes in the nucleus [155–157]. Their translocation to the cytosol, due to phosphorylation, inactivates these transcriptional targets [150]. Paradoxically, declines in the insulin/IGF-1 network were shown during both normal and premature aging [158].

The mTOR pathway is another major nutrient-sensing pathway with effects on aging [159]. Animal studies showed that genetic downregulation of mTOR or treatment with the mTOR inhibitor rapamycin extended longevity in yeast, worms, flies and mice [160, 161]. Rapamycin also improved and protected against age-related pathologies in mouse models of Alzheimer's disease, Parkinson's disease and cardiomyopathy [162, 163]. Along with these beneficial effects, several harming side-effects of mTOR inhibition, like impaired wound healing, insulin resistance and cataract, were shown in mice [161, 164].

AMP-activated kinase (AMPK) is a highly conserved nutrient and low-energy sensor [15, 165, 166]. Besides its functions in the maintenance of energy metabolism, it was suggested that AMPK coordinates a large signaling network of transcription factors [165, 167], including pathways involved in aging [166]. Several studies on model organisms demonstrated the crucial role of AMPK in the regulation of longevity in worms, flies and mice [166]. The responsiveness to AMPK signaling declines with age [168], contributing to many age-associated diseases [169].

The previously discussed sirtuins are part of the nutrient signaling pathway, as these NAD-dependent protein deacetylases respond to cellular low-energy states, cause epigenetic alterations and contribute to the aging process [15, 170].

##### Effect of H_2_S on nutrient sensing

Contributions of H_2_S signaling to several nutrient sensing pathways are described. H_2_S impairs the insulin/IGF-1 signaling pathway as it inhibits insulin secretion by pancreatic beta-cells and insulin secreting cell lines by stimulating ATP-sensitive potassium channels [171–173]. Administration of NaHS to cardiomyocytes increased glucose uptake by these cells [174, 175], increased the phosphorylation of several components of the insulin/IGF-1 signal pathway, like insulin receptor, PI3K and Akt [175], and improved glucose metabolism [174, 176]. H_2_S regulated vaso-relaxation in spontaneously hypertensive rats through the inhibition of FOXO1 and FOXO3 phosphorylation, which resulted in their nuclear translocation and their binding to target gene promotors [177]. H_2_S was also shown to function as an endogenous regulator of PTEN, the main antagonist of the PI3K-Akt axis in the insulin/IGF-1 signal pathway, by modifying PTEN activity through S-sulfhydration [178, 179]. Following incubation with H_2_S, oral keratinocyte stem cells increased their PTEN expression [180]. These studies indicate a regulatory role for H_2_S in the insulin/IGF-1 signaling network.

Administration of L-cysteine or Na_2_S to human U937 monocytes exposed to high-glucose, as a model for diabetes, increased cellular PIP3, AMPK phosphoryla-tion and PPARγ expression [181]. CSE inhibition prevented the L-cysteine-induced increase in PIP3 [181]. Various H_2_S-releasing compounds activated AMPK signaling, resulting in protective effects on cultured cells, tissues and whole organisms [182–186]. Interestingly, the well-established AMPK activator metformin, a widely prescribed insulin sensitizer and a first-line antidiabetic drug, increased H_2_S concentration in various tissues [187], indicating that H_2_S might mediate metformin's effects. H_2_S was shown to promote AMPK signaling and inhibit mTOR complex 1 activity in renal epithelial cells [188]. NaHS treatment blocked mTOR activity in Werner syndrome fibroblasts [147] and the human colon adenocarcinoma cell line HR-29 [189]. Contrary, NaHS treatment increased mTOR phosphorylation in reperfused hearts and concanavalin A-induced hepatitis [190, 191].

H_2_S also regulates the activity of the low-energy sensing sirtuins. Treatment with NaHS enhanced the SIRT1 deacetylase activity in human umbilical vein endothelial cells [119, 121]. In addition, increases in the expression of SIRT1 [119, 120], SIRT3 [180, 192] and SIRT6 [180, 193] were shown after treatment with H_2_S.

#### Mitochondrial dysfunction

Mitochondrial dysfunction and oxidative stress are thought to play an important role in aging by affecting intracellular signaling and interorganellar crosstalk [194–196]. Several age-related pathologies, like neurodegenerative diseases, diabetes, cancer, cellular senescence and impaired stem cell homeostasis were linked to ROS and dysfunctional mitochondria [197, 198]. Mild mitochondrial toxins, like metformin and resveratrol, retarded aging by inducing a low-energy state with increased AMP levels and AMPK activation [199] and by mediating the master antioxidant regulator Nrf2 [200]. These studies indicate that mild mito-chondrial stresses might be preventive against age-associated pathologies.

Mitochondria are the major producers of ROS, as its synthesis is an inevitable by-product of oxidative phosphorylation. The mitochondrial function declines with age, leading to increased electron leakage and ROS production and reduced ATP generation [196], which may, in turn, cause extra damage to the mitochondrial genome and further decline mitochondrial function. This vicious cycle, known as the mitochondrial free radical theory of aging [201], may lead to cellular energy depletion and ultimately to cell death [202, 203]. Since its proposal, numerous studies were performed to test the theory, generating inconsistent and conflicting results [204]. This led to a reconsideration of the role of ROS in aging [205]. In contrast to what was seen in severe mitochondrial stress, low physiological levels of intracellular ROS, maintained within a narrow range [206], have signaling functions in many cellular and systemic physiological processes [198, 207], inducing beneficial long-lasting metabolic and biochemical changes that may actually improve the cellular fitness [15, 197, 208, 209]. The phenomenon in which the exposure to low levels of a stressor induces compensatory biological processes, whereas higher levels disrupt homeostasis, is known as hormesis [209, 210]. Thus, dependent on its intracellular level, ROS can have both pathogenic, aging accelerating as well as lifespan increasing actions [208, 211, 212].

##### Effect of H_2_S on mitochondria

The effects of H_2_S on mitochondria are well described and its ability to reduce and modulate oxidative stress is considered to be one of the principal features of H_2_S in physiology. Several studies showed protective effects of H_2_S on mitochondrial function, as it increased the levels of antioxidants [213, 214], reduced the production of mitochondrial superoxides [215] and activated ROS-scavengers [216] and the anti-oxidative transcription factor Nrf2 [217, 218]. NaHS treatment stimulates the activities of superoxide dismutase and glutathione peroxidase and upregulated the expression of other antioxidants in human neuroblastoma cell line SH-SY5Y [143]. H_2_S improves mitochondrial ATP production in smooth muscle cells with impaired ATP production, especially following hypoxia [38]. Under stress conditions, cells can use H_2_S as an inorganic energy substrate for the mitochondrial respiratory chain to sustain ATP production [219–221]. It was proposed that high concentrations of H_2_S inhibit mitochondrial activity and protected organs against ischemia/reperfusion injury by reversible binding to cytochrome c oxidase, leading to hypometabolism, hypothermia and tissue preservation [79, 222–224].

As discussed previously, H_2_S protects the mitochondrial genome from damage and thereby preserves the mitochondrial integrity and the cellular energetics [38, 89, 125, 222]. Together these actions induced by H_2_S protect the integrity of mitochondria and prevent mitochondrial dysfunction.

#### Cellular senescence

The cell-cycle of damaged cells can be arrested and cells become senescent in order to prevent unrestricted growth [225, 226]. Senescent cells undergo characteristic phenotypic alterations, including activation of tumor-suppressors, epigenetic changes and changes to their secretome [15, 226, 227]. Cellular senescence was suggested to be a beneficial compensatory response to eliminate damaged, potentially oncogenic cells from tissues in order to replace them to re-establish cell numbers [15]. Experimental studies support this principle where a mild enhancement of the senescence pathways extended longevity in mice [228, 229] and elimination of the involved pathways was beneficial for mice with premature aging diseases [70, 230, 231].

Telomere attrition [232], DNA lesions [233], oxidative stress [234] and activated oncogenes [235, 236] are examples of stressors shown to induce senescence by the activation of the DNA damage response, which arrests cell-cycle through stabilization of p53 and transcriptional activation of p21 [226]. Other pathways, such as the p16^INK4a^/Rb and the p19^ARF^/p53 pathways, can also induce senescence, independent of the DNA damage response [226, 237]. These pathways were genetically linked to the highest number of age-related diseases [238] and correlated with the chronological age of essentially all tissues analyzed in both mice and humans [239, 240]. Other studies underscored the close relationship between metabolic changes and senescence [235, 241–243].

The altered secretome of senescent cells, with enhanced amounts of pro-inflammatory cytokines and chemokines, normally stimulates the innate immune system to eliminate senescent cells but may also contribute to or even accelerate the aging process when the turnover system becomes inefficient [15, 226, 227, 244]. The accumulation of senescent cells was shown in some, but not all, tissues with age demonstrating that this process became inefficient [15, 245]. During cell-cycle arrest, senescence can also be prevented by a process called assisted cycling, in which the cellular disabilities are mended [226, 246].

##### Effect of H_2_S on cellular senescence

Several lines of evidence indicate the involvement of H_2_S in cellular and organismal senescence. NaHS protects human umbilical vein endothelial cells against cellular senescence, potentially through the modulation of SIRT1 activity, and improves the function of senescent cells [119, 121]. Cellular aging can be delayed by decreasing oxidative stress. H_2_S protects against stress-induced cellular senescence by initiating the antioxidant responses, for example by S-sulfhydration of Keap1 leading to the activation of Nrf2 [218] and by inhibition of mitochondrial ROS production, by S-sulfhydration of p66Shc which prevents its phosphorylation and activation [247]. Others demonstrated that H_2_S induced the S-sulfhydration of MEK1, leading to PARP-1 activation and DNA damage repair, protecting cells from senescence [86]. Caloric restriction, reported to decelerate the biological aging process, maintained normal H_2_S levels, reduced oxidative stress-induced cellular senescence and promoted cellular cyto-protective systems [248]. In addition, deficiency of CSE in mouse embryonic fibroblasts led to early development of cellular senescence [218], under-scoring the regulative role of H_2_S signaling in senescence.

### Role of H_2_S in the integrative hallmarks of aging

#### Stem cell exhaustion

Stem cells regularly replace damaged or missing cells in tissues in order to maintain tissue homeostasis [249]. Most stem cells remain in their quiescent state and rarely enter cell-cycle in order to prevent the accumulation of damage during normal metabolic respiration and cell division [250]. However, not all damage can be prevented, resulting in a decline in stem cell function with age. As a consequence, their regenerative potential may be adversely affected, leading to organ failure and diseases of aging [250]. The previously discussed hallmarks are involved in the decline of stem cell function with age, as for example the accumulation of DNA damage [251] and telomere shortening [252, 253] were shown to attribute to stem cell exhaustion. In addition, stem cells are extremely sensitive to the loss of telomerase, which they normally express in order to maintain their genomic integrity [254, 255]. Stem cell attrition is thought not to be a direct result of the DNA damage itself but to be a product of the cellular responses to the damage, such as the activation of p53 that initiate DNA repair mechanisms and cell death programs [256, 257]. Studies also showed that stem cell function could be improved after calorie restriction [258, 259] or mTOR inhibition [259–261] in several tissues, resulting in improved proteostasis and affecting the energy sensing networks. A study in hematopoietic stem cells showed that epigenetic dysregulation during aging declined the regenerative potential and function of stem cells [262].

Besides the need to prevent functional decline of stem cells, organisms need to prevent the excessive proliferation of stem cells which could result in exhaustion of the stem cell compartment and accelerate aging [15]. Several physiological alterations observed during aging are thought to reflect an attempt of the organism to preserve the quiescence of stem cells [15].

##### Effect of H_2_S on stem cell maintenance

Several stem cell populations are affected by H_2_S. Endogenous H_2_S signaling maintains several biological functions of human periodontal ligament stem cells and neural stem cells, like the capacity to proliferate and differentiate [263–265]. Exogenous H_2_S may protect against neuronal decline normally observed after hypoxia [265]. In addition, NaHS increased and CSE inhibition decreased proliferation of human adipose tissue derived stem cells [266]. Bone marrow mesenchymal stem cells produce H_2_S in order to regulate their self-renewal and differentiation capacity [267]. Deficiencies in H_2_S signaling impaired stem cell function and bone homeostasis, which could be restored by H_2_S application [267]. Mesenchymal stem cells exposed to hypoxic conditions significantly decreased their H_2_S production and underwent apoptosis, whereas CSE overexpression protected against apoptosis [268]. Modulation of H_2_S signaling was suggested to be a potential therapeutic approach by which the viability of transplanted stem cells and the efficiency of cell-based therapy could be promoted [266, 268, 269].

Interestingly, the dedifferentiation of several cancer cells, a process in which cancer cells regain characteristics of undifferentiated stem cells, was characterized by the accumulation of H_2_S and the upregulation of H_2_S producing enzymes [270]. Reducing the H_2_S production in these cells reversed their ability to dedifferentiate, whereas the accumulation of H_2_S induced their dedifferentiation [270]. Altogether, these studies show that stem cell maintenance and H_2_S signaling are connected.

#### Altered intercellular communication

Whereas the previously discussed hallmarks of aging mainly focused on age-associated intracellular alterations, the interaction between cells also changes with age. As a result of aging, the extracellular environment of cells may change, altering their intercellular communication [15]. The changed secretome of senescent cells is an example of such an alteration in the paracrine interaction between cells [15, 226, 227, 244]. Senescent cells were also described to induce senescence in neighboring cells via juxtacrine signaling and processes involving ROS [271]. Alterations on several other levels of intercellular communication, like endocrine, neuroendocrine or neuronal signaling [150, 272–274], during aging have been described [15]. The age-associated alterations in the insulin/IGF-1 network are examples of how the neuroendocrine communication can be altered with age. Another important example of altered intercellular communication is ‘inflammaging’, in which mammalian aging is accompanied by a pro-inflammatory phenotype [275]. Some age-related pathologies, like obesity, type 2 diabetes [152] and atherosclerosis [276], were associated with inflammation. Numerous age-associated alterations, such as the pro-inflammatory secretome of senescent cells, the activation of NF-κB signaling and a failing autophagy response during aging, contribute to inflammaging [196, 275]. The function of the adaptive immune system declines with age due to immuno-senescence leading to impaired clearance of infectious agents and infected, damaged or senescent cells, which may, in turn, aggravate the aging phenotype [15, 277]. Sirtuins may also contribute to this phenotype by changing the expression of inflammatory genes [278].

The age-associated alterations in intercellular communication also explain the interorgan coordination of aging, in which lifespan-extending manipulations in one tissue were described to retard the aging process in other tissues [279–282]. Manipulation of the gut microbiome was also suggested to retard aging as it may affect the efficiency and function of the host immune system and exert systemic metabolic effects [15, 283, 284].

##### Effect of H_2_S on intercellular communication

Several studies showed that H_2_S treatment alters intercellular communication. Hydrogen sulfide was proposed to mediate inflammation, however both pro- and anti-inflammatory actions are described [8]. At low, physiological concentrations, H_2_S is predominantly anti-inflammatory, whereas high H_2_S concentrations may promote inflammation [28, 285]. Pro-inflammatory cytokines increase endogenous H_2_S production in chondrocytes and mesenchymal progenitor cells [286], whereas L-cysteine or NaHS administration decreased NF-κB phosphorylation and reduced the secretion of pro-inflammatory cytokines in human U937 monocytes treated with high-glucose [181]. Exogenous H_2_S protects H9c2 cardiac cells against high glucose-induced inflammation and injury by inhibiting the NF-κB/IL-1β pathway [287]. Consistently, NaHS decreased the secretion of pro-inflammatory cytokines in mice with severe hepatic ischemia and reperfusion injury, via mechanisms involving Nrf2 [288]. Neuroinflammation and vascular inflammation were modulated by H_2_S, in part through the activation of AMPK and the reduction of oxidative stress by H_2_S [181, 183]. Beside the effects of H_2_S on inflammation, H_2_S depresses gap junction intercellular communication, which inhibited human platelet aggregation *in vitro* [289]. Diffusion of H_2_S from an H_2_S-producing cell into neighboring cells affects ion management and intracellular processes [8, 146, 290]. H_2_S regulates various ion channels and transporters by sulfhydrating specific cysteine residues of subunits of these protein complexes [8, 146, 290].

Several calcium and potassium channels associated with biological processes, such as cardiac contraction, sensory transduction, inflammation and neuro-protection, were shown to be manipulated by H_2_S [8, 146, 290]. These effects of H_2_S have also been observed in the cerebral microcirculation in response to hypoxia, where H_2_S produced in astrocytes causes vasodilatation by diffusion into the contractile pericytes that surround the arterioles [291]. Impaired H_2_S production might thus result in vascular dysfunction and other pathophysiologies [8, 146]. Taken together, these studies demonstrate that H2S may have anti-aging properties by altering intercellular communication.

## PERSPECTIVES

**Figure 3:**
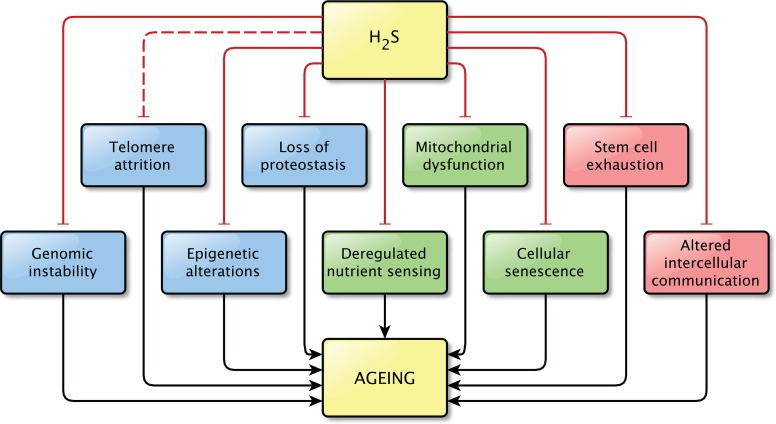
Overview of the effects of physiological levels of H_2_S on the Hallmarks of Aging Hydrogen sulfide affects at least one pathway involved in almost all hallmarks of aging. A direct effect of H_2_S on pathways involved in telomere attrition was not shown, however the effects of H_2_S on genome stability might also be beneficial for telomere maintenance, by protecting the integrity of the genome. This is indicated by the interrupted line between H_2_S and telomere attrition. Physiological levels of H_2_S were shown to prevent the dysregulation of the pathways associated with aging.

The precise relationship between H_2_S and aging still remains unknown. However, the findings discussed in this review strongly support the idea that H_2_S plays a role in the process of aging and in age-related pathologies, as direct effects on pathways related to aging were shown in all but one hallmarks of aging (Figure 3). It is possible that not all effects described here are important in the light of aging, but together they indicate that the contribution of H_2_S signaling to normal physiology and to pathophysiology is not to be neglected.

Treatment with H_2_S or influencing the transsulfuration pathways may become an intervention to prevent, delay or reverse aging and accompanying symptoms and pathologies. However, both beneficial as well as detrimental effects of H_2_S application were shown in several hallmarks, indicating that administration of H_2_S dictates great caution. The underlying pathways of both the aging process and the (patho-) physiological processes of H_2_S signaling need to be studied more extensively. Combining these studies may shed new lights on the role of H_2_S in aging is needed in order to determine the possible preventive and therapeutic potential of H_2_S on the process of aging.

## References

[1] Wang H, Dwyer-Lindgren L, Lofgren KT, Rajaratnam JK, Marcus JR, Levin-Rector A, Levitz CE, Lopez AD, Murray CJ (2012). Age-specific and sex-specific mortality in 187 countries, 1970-2010: a systematic analysis for the Global Burden of Disease Study 2010. Lancet.

[2] Newgard CB, Sharpless NE (2013). Coming of age: molecular drivers of aging and therapeutic opportunities. J Clin Invest.

[3] Qabazard B, Stürzenbaum SR (2015). H2S: A new approach to lifespan enhancement and healthy ageing?. Handb Exp Pharmacol.

[4] Niccoli T, Partridge L (2012). Ageing as a risk factor for disease. Curr Biol.

[5] Dröge W (2002). Aging-related changes in the thiol/disulfide redox state: implications for the use of thiol antioxidants. Exp Gerontol.

[6] Miller DL, Roth MB (2007). Hydrogen sulfide increases thermotolerance and lifespan in Caenorhabditis elegans. Proc Natl Acad Sci USA.

[7] Qabazard B, Li L, Gruber J, Peh MT, Ng LF, Kumar SD, Rose P, Tan CH, Dymock BW, Wei F, Swain SC, Halliwell B, Stürzenbaum SR, Moore PK (2014). Hydrogen sulfide is an endogenous regulator of aging in Caenorhabditis elegans. Antioxid Redox Signal.

[8] Wang R (2012). Physiological implications of hydrogen sulfide: a whiff exploration that blossomed. Physiol Rev.

[9] Chen YH, Yao WZ, Geng B, Ding YL, Lu M, Zhao MW, Tang CS (2005). Endogenous hydrogen sulfide in patients with COPD. Chest.

[10] Zhang Y, Tang ZH, Ren Z, Qu SL, Liu MH, Liu LS, Jiang ZS (2013). Hydrogen sulfide, the next potent preventive and therapeutic agent in aging and age-associated diseases. Mol Cell Biol.

[11] Golden TR, Melov S (2007). Gene expression changes associated with aging in C. elegans. WormBook.

[12] Gems D, Partridge L (2013). Genetics of longevity in model organisms: debates and paradigm shifts. Annu Rev Physiol.

[13] Kirkwood TB (2005). Understanding the odd science of aging. Cell.

[14] Vijg J, Campisi J (2008). Puzzles, promises and a cure for ageing. Nature.

[15] López-Otín C, Blasco MA, Partridge L, Serrano M, Kroemer G (2013). The hallmarks of aging. Cell.

[16] Wang R (2002). Two's company, three's a crowd: can H2S be the third endogenous gaseous transmitter?. FASEB J.

[17] Wang R (2014). Gasotransmitters: growing pains and joys. Trends Biochem Sci.

[18] Heinemann SH, Hoshi T, Westerhausen M, Schiller A (2014). Carbon monoxide--physiology, detection and controlled release. Chem Commun (Camb).

[19] Kelm M (1999). Nitric oxide metabolism and breakdown. Biochim Biophys Acta.

[20] Kajimura M, Fukuda R, Bateman RM, Yamamoto T, Suematsu M (2010). Interactions of multiple gas-transducing systems: hallmarks and uncertainties of CO, NO, and H2S gas biology. Antioxid Redox Signal.

[21] Snijder PM, van den Berg E, Whiteman M, Bakker SJ, Leuvenink HG, van Goor H (2013). Emerging role of gasotransmitters in renal transplantation. Am J Transplant.

[22] Filipovic MR, Eberhardt M, Prokopovic V, Mijuskovic A, Orescanin-Dusic Z, Reeh P, Ivanovic-Burmazovic I (2013). Beyond H2S and NO interplay: hydrogen sulfide and nitroprusside react directly to give nitroxyl (HNO). A new pharmacological source of HNO. J Med Chem.

[23] Zhao W, Zhang J, Lu Y, Wang R (2001). The vasorelaxant effect of H(2)S as a novel endogenous gaseous K(ATP) channel opener. EMBO J.

[24] Altaany Z, Yang G, Wang R (2013). Crosstalk between hydrogen sulfide and nitric oxide in endothelial cells. J Cell Mol Med.

[25] Beauchamp RO, Bus JS, Popp JA, Boreiko CJ, Andjelkovich DA, Leber P (1984). A critical review of the literature on hydrogen sulfide toxicity. Crit Rev Toxicol.

[26] Abe K, Kimura H (1996). The possible role of hydrogen sulfide as an endogenous neuromodulator. J Neurosci.

[27] Hosoki R, Matsuki N, Kimura H (1997). The possible role of hydrogen sulfide as an endogenous smooth muscle relaxant in synergy with nitric oxide. Biochem Biophys Res Commun.

[28] Szabó C (2007). Hydrogen sulphide and its therapeutic potential. Nat Rev Drug Discov.

[29] Li Q, Lancaster JR (2013). Chemical foundations of hydrogen sulfide biology. Nitric Oxide.

[30] Bełtowski J (2015). Hydrogen sulfide in pharmacology and medicine--An update. Pharmacol Rep.

[31] Li L, Hsu A, Moore PK (2009). Actions and interactions of nitric oxide, carbon monoxide and hydrogen sulphide in the cardiovascular system and in inflammation--a tale of three gases!. Pharmacol Ther.

[32] Whitfield NL, Kreimier EL, Verdial FC, Skovgaard N, Olson KR (2008). Reappraisal of H2S/sulfide concentration in vertebrate blood and its potential significance in ischemic preconditioning and vascular signaling. Am J Physiol Regul Integr Comp Physiol.

[33] Searcy DG, Lee SH (1998). Sulfur reduction by human erythrocytes. J Exp Zool.

[34] Benavides GA, Squadrito GL, Mills RW, Patel HD, Isbell TS, Patel RP, Darley-Usmar VM, Doeller JE, Kraus DW (2007). Hydrogen sulfide mediates the vasoactivity of garlic. Proc Natl Acad Sci USA.

[35] Kamoun P (2004). Endogenous production of hydrogen sulfide in mammals. Amino Acids.

[36] Kabil O, Banerjee R (2014). Enzymology of H2S biogenesis, decay and signaling. Antioxid Redox Signal.

[37] Teng H, Wu B, Zhao K, Yang G, Wu L, Wang R (2013). Oxygen-sensitive mitochondrial accumulation of cystathionine β-synthase mediated by Lon protease. Proc Natl Acad Sci USA.

[38] Fu M, Zhang W, Wu L, Yang G, Li H, Wang R (2012). Hydrogen sulfide (H2S) metabolism in mitochondria and its regulatory role in energy production. Proc Natl Acad Sci USA.

[39] Koning AM, Frenay AR, Leuvenink HG, van Goor H (2015). Hydrogen sulfide in renal physiology, disease and transplantation--the smell of renal protection. Nitric Oxide.

[40] Nagahara N, Ito T, Kitamura H, Nishino T (1998). Tissue and subcellular distribution of mercaptopyruvate sulfurtransferase in the rat: confocal laser fluorescence and immunoelectron microscopic studies combined with biochemical analysis. Histochem Cell Biol.

[41] Kimura H (2014). The physiological role of hydrogen sulfide and beyond. Nitric Oxide.

[42] Singh S, Padovani D, Leslie RA, Chiku T, Banerjee R (2009). Relative contributions of cystathionine beta-synthase and gamma-cystathionase to H2S biogenesis via alternative trans-sulfuration reactions. J Biol Chem.

[43] Shibuya N, Koike S, Tanaka M, Ishigami-Yuasa M, Kimura Y, Ogasawara Y, Fukui K, Nagahara N, Kimura H (2013). A novel pathway for the production of hydrogen sulfide from D-cysteine in mammalian cells. Nat Commun.

[44] Mikami Y, Shibuya N, Kimura Y, Nagahara N, Ogasawara Y, Kimura H (2011). Thioredoxin and dihydrolipoic acid are required for 3-mercaptopyruvate sulfurtransferase to produce hydrogen sulfide. Biochem J.

[45] Koj A, Frendo J, Janik Z (1967). [35S]thiosulphate oxidation by rat liver mitochondria in the presence of glutathione. Biochem J.

[46] Beerens H, Romond C (1977). Sulfate-reducing anaerobic bacteria in human feces. Am J Clin Nutr.

[47] Pitcher MC, Beatty ER, Harris RM, Waring RH, Cummings JH (1998). Sulfur metabolism in ulcerative colitis: investigation of detoxification enzymes in peripheral blood. Dig Dis Sci.

[48] Florin T, Neale G, Gibson GR, Christl SU, Cummings JH (1991). Metabolism of dietary sulphate: absorption and excretion in humans. Gut.

[49] Suarez F, Furne J, Springfield J, Levitt M (1998). Production and elimination of sulfur-containing gases in the rat colon. Am J Physiol.

[50] Furne J, Springfield J, Koenig T, DeMaster E, Levitt MD (2001). Oxidation of hydrogen sulfide and methanethiol to thiosulfate by rat tissues: a specialized function of the colonic mucosa. Biochem Pharmacol.

[51] Macfarlane S, Steed H, Macfarlane GT (2009). Intestinal bacteria and inflammatory bowel disease. Crit Rev Clin Lab Sci.

[52] Rowan FE, Docherty NG, Coffey JC, O'Connell PR (2009). Sulphate-reducing bacteria and hydrogen sulphide in the aetiology of ulcerative colitis. Br J Surg.

[53] Medani M, Collins D, Docherty NG, Baird AW, O'Connell PR, Winter DC (2011). Emerging role of hydrogen sulfide in colonic physiology and pathophysiology. Inflamm Bowel Dis.

[54] Kabil O, Banerjee R (2012). Characterization of patient mutations in human persulfide dioxygenase (ETHE1) involved in H2S catabolism. J Biol Chem.

[55] Tiranti V, D'Adamo P, Briem E, Ferrari G, Mineri R, Lamantea E, Mandel H, Balestri P, Garcia-Silva MT, Vollmer B, Rinaldo P, Hahn SH, Leonard J (2004). Ethylmalonic encephalopathy is caused by mutations in ETHE1, a gene encoding a mitochondrial matrix protein. Am J Hum Genet.

[56] Tiranti V, Viscomi C, Hildebrandt T, Di Meo I, Mineri R, Tiveron C, Levitt MD, Prelle A, Fagiolari G, Rimoldi M, Zeviani M (2009). Loss of ETHE1, a mitochondrial dioxygenase, causes fatal sulfide toxicity in ethylmalonic encephalopathy. Nat Med.

[57] Di Meo I, Fagiolari G, Prelle A, Viscomi C, Zeviani M, Tiranti V (2011). Chronic exposure to sulfide causes accelerated degradation of cytochrome c oxidase in ethylmalonic encephalopathy. Antioxid Redox Signal.

[58] Viscomi C, Burlina AB, Dweikat I, Savoiardo M, Lamperti C, Hildebrandt T, Tiranti V, Zeviani M (2010). Combined treatment with oral metronidazole and N-acetylcysteine is effective in ethylmalonic encephalopathy. Nat Med.

[59] Mustafa AK, Sikka G, Gazi SK, Steppan J, Jung SM, Bhunia AK, Barodka VM, Gazi FK, Barrow RK, Wang R, Amzel LM, Berkowitz DE, Snyder SH (2011). Hydrogen sulfide as endothelium-derived hyperpolarizing factor sulfhydrates potassium channels. Circ Res.

[60] Yang G, Wu L, Jiang B, Yang W, Qi J, Cao K, Meng Q, Mustafa AK, Mu W, Zhang S, Snyder SH, Wang R (2008). H2S as a physiologic vasorelaxant: hypertension in mice with deletion of cystathionine gamma-lyase. Science.

[61] Hoeijmakers JH (2009). DNA damage, aging, and cancer. N Engl J Med.

[62] Lord CJ, Ashworth A (2012). The DNA damage response and cancer therapy. Nature.

[63] Baker DJ, Dawlaty MM, Wijshake T, Jeganathan KB, Malureanu L, van Ree JH, Crespo-Diaz R, Reyes S, Seaburg L, Shapiro V, Behfar A, Terzic A, van de Sluis B, van Deursen JM (2013). Increased expression of BubR1 protects against aneuploidy and cancer and extends healthy lifespan. Nat Cell Biol.

[64] Linnane AW, Marzuki S, Ozawa T, Tanaka M (1989). Mitochondrial DNA mutations as an important contributor to ageing and degenerative diseases. Lancet.

[65] Kazak L, Reyes A, Holt IJ (2012). Minimizing the damage: repair pathways keep mitochondrial DNA intact. Nat Rev Mol Cell Biol.

[66] Park CB, Larsson NG (2011). Mitochondrial DNA mutations in disease and aging. J Cell Biol.

[67] Dechat T, Pfleghaar K, Sengupta K, Shimi T, Shumaker DK, Solimando L, Goldman RD (2008). Nuclear lamins: major factors in the structural organization and function of the nucleus and chromatin. Genes Dev.

[68] Ragnauth CD, Warren DT, Liu Y, McNair R, Tajsic T, Figg N, Shroff R, Skepper J, Shanahan CM (2010). Prelamin A acts to accelerate smooth muscle cell senescence and is a novel biomarker of human vascular aging. Circulation.

[69] Scaffidi P, Misteli T (2006). Lamin A-dependent nuclear defects in human aging. Science.

[70] Varela I, Cadiñanos J, Pendás AM, Gutiérrez-Fernández A, Folgueras AR, Sánchez LM, Zhou Z, Rodríguez FJ, Stewart CL, Vega JA, Tryggvason K, Freije JM, López-Otín C (2005). Accelerated ageing in mice deficient in Zmpste24 protease is linked to p53 signalling activation. Nature.

[71] Mariño G, Ugalde AP, Fernández AF, Osorio FG, Fueyo A, Freije JM, López-Otín C (2010). Insulin-like growth factor 1 treatment extends longevity in a mouse model of human premature aging by restoring somatotroph axis function. Proc Natl Acad Sci USA.

[72] Espada J, Varela I, Flores I, Ugalde AP, Cadiñanos J, Pendás AM, Stewart CL, Tryggvason K, Blasco MA, Freije JM, López-Otín C (2008). Nuclear envelope defects cause stem cell dysfunction in premature-aging mice. J Cell Biol.

[73] Scaffidi P, Misteli T (2008). Lamin A-dependent misregulation of adult stem cells associated with accelerated ageing. Nat Cell Biol.

[74] Osorio FG, Bárcena C, Soria-Valles C, Ramsay AJ, de Carlos F, Cobo J, Fueyo A, Freije JM, López-Otín C (2012). Nuclear lamina defects cause ATM-dependent NF-κB activation and link accelerated aging to a systemic inflammatory response. Genes Dev.

[75] Attene-Ramos MS, Wagner ED, Plewa MJ, Gaskins HR (2006). Evidence that hydrogen sulfide is a genotoxic agent. Mol Cancer Res.

[76] Baskar R, Li L, Moore PK (2007). Hydrogen sulfide-induces DNA damage and changes in apoptotic gene expression in human lung fibroblast cells. FASEB J.

[77] Deplancke B, Finster K, Graham WV, Collier CT, Thurmond JE, Gaskins HR (2003). Gastrointestinal and microbial responses to sulfate-supplemented drinking water in mice. Exp Biol Med (Maywood).

[78] Magee EA, Richardson CJ, Hughes R, Cummings JH (2000). Contribution of dietary protein to sulfide production in the large intestine: an in vitro and a controlled feeding study in humans. Am J Clin Nutr.

[79] Jha S, Calvert JW, Duranski MR, Ramachandran A, Lefer DJ (2008). Hydrogen sulfide attenuates hepatic ischemia-reperfusion injury: role of antioxidant and antiapoptotic signaling. Am J Physiol Heart Circ Physiol.

[80] Jones DP (2008). Radical-free biology of oxidative stress. Am J Physiol Cell Physiol.

[81] Furne J, Saeed A, Levitt MD (2008). Whole tissue hydrogen sulfide concentrations are orders of magnitude lower than presently accepted values. Am J Physiol Regul Integr Comp Physiol.

[82] Wedmann R, Bertlein S, Macinkovic I, Böltz S, Miljkovic JL, Muñoz LE, Herrmann M, Filipovic MR (2014). Working with “H2S”: facts and apparent artifacts. Nitric Oxide.

[83] Nagy P, Palinkas Z, Nagy A, Budai B, Toth I, Vasas A (2014). Chemical aspects of hydrogen sulfide measurements in physiological samples. Biochim Biophys Acta.

[84] Vitvitsky V, Kabil O, Banerjee R (2012). High turnover rates for hydrogen sulfide allow for rapid regulation of its tissue concentrations. Antioxid Redox Signal.

[85] DeLeon ER, Stoy GF, Olson KR (2012). Passive loss of hydrogen sulfide in biological experiments. Anal Biochem.

[86] Zhao K, Ju Y, Li S, Altaany Z, Wang R, Yang G (2014). S-sulfhydration of MEK1 leads to PARP-1 activation and DNA damage repair. EMBO Rep.

[87] Pan Y, Ye S, Yuan D, Zhang J, Bai Y, Shao C (2014). Hydrogen sulfide (H2S)/cystathionine γ-lyase (CSE) pathway contributes to the proliferation of hepatoma cells. Mutat Res.

[88] Guzmán R, Campos C, Yuguero R, Masegù C, Gil P, Moragón AC (2015). Protective effect of sulfurous water in peripheral blood mononuclear cells of Alzheimer's disease patients. Life Sci.

[89] Szczesny B, Módis K, Yanagi K, Coletta C, Le Trionnaire S, Perry A, Wood ME, Whiteman M, Szabo C (2014). AP39, a novel mitochondria-targeted hydrogen sulfide donor, stimulates cellular bioenergetics, exerts cyto-protective effects and protects against the loss of mitochondrial DNA integrity in oxidatively stressed endothelial cells in vitro. Nitric Oxide.

[90] Wu D, Hu Q, Liu X, Pan L, Xiong Q, Zhu YZ (2015). Hydrogen sulfide protects against apoptosis under oxidative stress through SIRT1 pathway in H9c2 cardiomyocytes. Nitric Oxide.

[91] Blackburn EH, Greider CW, Szostak JW (2006). Telomeres and telomerase: the path from maize, Tetrahymena and yeast to human cancer and aging. Nat Med.

[92] Aubert G, Lansdorp PM (2008). Telomeres and aging. Physiol Rev.

[93] Blasco MA (2007). Telomere length, stem cells and aging. Nat Chem Biol.

[94] Fumagalli M, Rossiello F, Clerici M, Barozzi S, Cittaro D, Kaplunov JM, Bucci G, Dobreva M, Matti V, Beausejour CM, Herbig U, Longhese MP, d'Adda di Fagagna F (2012). Telomeric DNA damage is irreparable and causes persistent DNA-damage-response activation. Nat Cell Biol.

[95] Hewitt G, Jurk D, Marques FD, Correia-Melo C, Hardy T, Gackowska A, Anderson R, Taschuk M, Mann J, Passos JF (2012). Telomeres are favoured targets of a persistent DNA damage response in ageing and stress-induced senescence. Nat Commun.

[96] Jaskelioff M, Muller FL, Paik JH, Thomas E, Jiang S, Adams AC, Sahin E, Kost-Alimova M, Protopopov A, Cadiñanos J, Horner JW, Maratos-Flier E, Depinho RA (2011). Telomerase reactivation reverses tissue degeneration in aged telomerase-deficient mice. Nature.

[97] Benayoun BA, Pollina EA, Brunet A (2015). Epigenetic regulation of ageing: linking environmental inputs to genomic stability. Nat Rev Mol Cell Biol.

[98] Ben-Avraham D (2015). Epigenetics of aging. Adv Exp Med Biol.

[99] Weber CM, Henikoff S (2014). Histone variants: dynamic punctuation in transcription. Genes Dev.

[100] Bernstein BE, Meissner A, Lander ES (2007). The mammalian epigenome. Cell.

[101] Margueron R, Trojer P, Reinberg D (2005). The key to development: interpreting the histone code?. Curr Opin Genet Dev.

[102] Bird A (2002). DNA methylation patterns and epigenetic memory. Genes Dev.

[103] Fraga MF, Esteller M (2007). Epigenetics and aging: the targets and the marks. Trends Genet.

[104] Han S, Brunet A (2012). Histone methylation makes its mark on longevity. Trends Cell Biol.

[105] Guarente L (2011). Sirtuins, aging, and metabolism. Cold Spring Harb Symp Quant Biol.

[106] Sharma S, Kelly TK, Jones PA (2010). Epigenetics in cancer. Carcinogenesis.

[107] Hernandez DG, Nalls MA, Gibbs JR, Arepalli S, van der Brug M, Chong S, Moore M, Longo DL, Cookson MR, Traynor BJ, Singleton AB (2011). Distinct DNA methylation changes highly correlated with chronological age in the human brain. Hum Mol Genet.

[108] Heyn H, Li N, Ferreira HJ, Moran S, Pisano DG, Gomez A, Diez J, Sanchez-Mut JV, Setien F, Carmona FJ, Puca AA, Sayols S, Pujana MA (2012). Distinct DNA methylomes of newborns and centenarians. Proc Natl Acad Sci USA.

[109] Maegawa S, Hinkal G, Kim HS, Shen L, Zhang L, Zhang J, Zhang N, Liang S, Donehower LA, Issa JP (2010). Widespread and tissue specific age-related DNA methylation changes in mice. Genome Res.

[110] Bell JT, Tsai PC, Yang TP, Pidsley R, Nisbet J, Glass D, Mangino M, Zhai G, Zhang F, Valdes A, Shin SY, Dempster EL, Murray RM, MuTHER Consortium (2012). Epigenome-wide scans identify differentially methylated regions for age and age-related phenotypes in a healthy ageing population. PLoS Genet.

[111] Pegoraro G, Kubben N, Wickert U, Göhler H, Hoffmann K, Misteli T (2009). Ageing-related chromatin defects through loss of the NURD complex. Nat Cell Biol.

[112] Pollina EA, Brunet A (2011). Epigenetic regulation of aging stem cells. Oncogene.

[113] Larson K, Yan SJ, Tsurumi A, Liu J, Zhou J, Gaur K, Guo D, Eickbush TH, Li WX (2012). Heterochromatin formation promotes longevity and represses ribosomal RNA synthesis. PLoS Genet.

[114] Rios EC, Szczesny B, Soriano FG, Olah G, Szabo C (2015). Hydrogen sulfide attenuates cytokine production through the modulation of chromatin remodeling. Int J Mol Med.

[115] Li L, Liu D, Bu D, Chen S, Wu J, Tang C, Du J, Jin H (2013). Brg1-dependent epigenetic control of vascular smooth muscle cell proliferation by hydrogen sulfide. Biochim Biophys Acta.

[116] Druesne N, Pagniez A, Mayeur C, Thomas M, Cherbuy C, Duée PH, Martel P, Chaumontet C (2004). Diallyl disulfide (DADS) increases histone acetylation and p21(waf1/cip1) expression in human colon tumor cell lines. Carcinogenesis.

[117] Arunkumar A, Vijayababu MR, Gunadharini N, Krishnamoorthy G, Arunakaran J (2007). Induction of apoptosis and histone hyperacetylation by diallyl disulfide in prostate cancer cell line PC-3. Cancer Lett.

[118] Bhuiyan AI, Papajani VT, Paci M, Melino S (2015). Glutathione-garlic sulfur conjugates: slow hydrogen sulfide releasing agents for therapeutic applications. Molecules.

[119] Zheng M, Qiao W, Cui J, Liu L, Liu H, Wang Z, Yan C (2014). Hydrogen sulfide delays nicotinamide-induced premature senescence via upregulation of SIRT1 in human umbilical vein endothelial cells. Mol Cell Biochem.

[120] Li X, Zhang KY, Zhang P, Chen LX, Wang L, Xie M, Wang CY, Tang XQ (2014). Hydrogen sulfide inhibits formaldehyde-induced endoplasmic reticulum stress in PC12 cells by upregulation of SIRT-1. PLoS One.

[121] Suo R, Zhao ZZ, Tang ZH, Ren Z, Liu X, Liu LS, Wang Z, Tang CK, Wei DH, Jiang ZS (2013). Hydrogen sulfide prevents H2O2-induced senescence in human umbilical vein endothelial cells through SIRT1 activation. Mol Med Rep.

[122] Tanno M, Kuno A, Yano T, Miura T, Hisahara S, Ishikawa S, Shimamoto K, Horio Y (2010). Induction of manganese superoxide dismutase by nuclear translocation and activation of SIRT1 promotes cell survival in chronic heart failure. J Biol Chem.

[123] Li JJ, Li Q, Du HP, Wang YL, You SJ, Wang F, Xu XS, Cheng J, Cao YJ, Liu CF, Hu LF (2015). Homocysteine triggers inflammatory responses in macrophages through inhibiting CSE-H2S signaling via DNA hypermethylation of CSE promoter. Int J Mol Sci.

[124] Kamat PK, Kalani A, Tyagi SC, Tyagi N (2015). Hydrogen sulfide epigenetically attenuates homocysteine-induced mitochondrial toxicity mediated through NMDA receptor in mouse brain endothelial (bEnd3) cells. J Cell Physiol.

[125] Li S, Yang G (2015). Hydrogen sulfide maintains mitochondrial DNA replication via demethylation of TFAM. Antioxid Redox Signal.

[126] Koga H, Kaushik S, Cuervo AM (2011). Protein homeostasis and aging: the importance of exquisite quality control. Ageing Res Rev.

[127] Powers ET, Morimoto RI, Dillin A, Kelly JW, Balch WE (2009). Biological and chemical approaches to diseases of proteostasis deficiency. Annu Rev Biochem.

[128] Chiti F, Dobson CM (2006). Protein misfolding, functional amyloid, and human disease. Annu Rev Biochem.

[129] Labbadia J, Morimoto RI (2015). The biology of proteostasis in aging and disease. Annu Rev Biochem.

[130] Hartl FU, Bracher A, Hayer-Hartl M (2011). Molecular chaperones in protein folding and proteostasis. Nature.

[131] Morrow G, Samson M, Michaud S, Tanguay RM (2004). Overexpression of the small mitochondrial Hsp22 extends Drosophila life span and increases resistance to oxidative stress. FASEB J.

[132] Walker GA, Lithgow GJ (2003). Lifespan extension in C. elegans by a molecular chaperone dependent upon insulin-like signals. Aging Cell.

[133] Min JN, Whaley RA, Sharpless NE, Lockyer P, Portbury AL, Patterson C (2008). CHIP deficiency decreases longevity, with accelerated aging phenotypes accompanied by altered protein quality control. Mol Cell Biol.

[134] Calderwood SK, Murshid A, Prince T (2009). The shock of aging: molecular chaperones and the heat shock response in longevity and aging--a mini-review. Gerontology.

[135] Mizushima N, Levine B, Cuervo AM, Klionsky DJ (2008). Autophagy fights disease through cellular self-digestion. Nature.

[136] Rubinsztein DC, Mariño G, Kroemer G (2011). Autophagy and aging. Cell.

[137] Tomaru U, Takahashi S, Ishizu A, Miyatake Y, Gohda A, Suzuki S, Ono A, Ohara J, Baba T, Murata S, Tanaka K, Kasahara M (2012). Decreased proteasomal activity causes age-related phenotypes and promotes the development of metabolic abnormalities. Am J Pathol.

[138] Bjedov I, Toivonen JM, Kerr F, Slack C, Jacobson J, Foley A, Partridge L (2010). Mechanisms of life span extension by rapamycin in the fruit fly Drosophila melanogaster. Cell Metab.

[139] Eisenberg T, Knauer H, Schauer A, Büttner S, Ruckenstuhl C, Carmona-Gutierrez D, Ring J, Schroeder S, Magnes C, Antonacci L, Fussi H, Deszcz L, Hartl R (2009). Induction of autophagy by spermidine promotes longevity. Nat Cell Biol.

[140] Liu G, Rogers J, Murphy CT, Rongo C (2011). EGF signalling activates the ubiquitin proteasome system to modulate C. elegans lifespan. EMBO J.

[141] Kruegel U, Robison B, Dange T, Kahlert G, Delaney JR, Kotireddy S, Tsuchiya M, Tsuchiyama S, Murakami CJ, Schleit J, Sutphin G, Carr D, Tar K (2011). Elevated proteasome capacity extends replicative lifespan in Saccharomyces cerevisiae. PLoS Genet.

[142] Talaei F, Van Praag VM, Shishavan MH, Landheer SW, Buikema H, Henning RH (2014). Increased protein aggregation in Zucker diabetic fatty rat brain: identification of key mechanistic targets and the therapeutic application of hydrogen sulfide. BMC Cell Biol.

[143] Liu YY, Nagpure BV, Wong PT, Bian JS (2013). Hydrogen sulfide protects SH-SY5Y neuronal cells against d-galactose induced cell injury by suppression of advanced glycation end products formation and oxidative stress. Neurochem Int.

[144] Fawcett EM, Hoyt JM, Johnson JK, Miller DL (2015). Hypoxia disrupts proteostasis in Caenorhabditis elegans. Aging Cell.

[145] Yang Z, Yang C, Xiao L, Liao X, Lan A, Wang X, Guo R, Chen P, Hu C, Feng J (2011). Novel insights into the role of HSP90 in cytoprotection of H2S against chemical hypoxia-induced injury in H9c2 cardiac myocytes. Int J Mol Med.

[146] Paul BD, Snyder SH (2012). H2S signalling through protein sulfhydration and beyond. Nat Rev Mol Cell Biol.

[147] Talaei F, van Praag VM, Henning RH (2013). Hydrogen sulfide restores a normal morphological phenotype in Werner syndrome fibroblasts, attenuates oxidative damage and modulates mTOR pathway. Pharmacol Res.

[148] Colman RJ, Anderson RM, Johnson SC, Kastman EK, Kosmatka KJ, Beasley TM, Allison DB, Cruzen C, Simmons HA, Kemnitz JW, Weindruch R (2009). Caloric restriction delays disease onset and mortality in rhesus monkeys. Science.

[149] Kenyon CJ (2010). The genetics of ageing. Nature.

[150] Russell SJ, Kahn CR (2007). Endocrine regulation of ageing. Nat Rev Mol Cell Biol.

[151] Fontana L, Partridge L, Longo VD (2010). Extending healthy life span--from yeast to humans. Science.

[152] Barzilai N, Huffman DM, Muzumdar RH, Bartke A (2012). The critical role of metabolic pathways in aging. Diabetes.

[153] Suh Y, Atzmon G, Cho MO, Hwang D, Liu B, Leahy DJ, Barzilai N, Cohen P (2008). Functionally significant insulin-like growth factor I receptor mutations in centenarians. Proc Natl Acad Sci USA.

[154] Kojima T, Kamei H, Aizu T, Arai Y, Takayama M, Nakazawa S, Ebihara Y, Inagaki H, Masui Y, Gondo Y, Sakaki Y, Hirose N (2004). Association analysis between longevity in the Japanese population and polymorphic variants of genes involved in insulin and insulin-like growth factor 1 signaling pathways. Exp Gerontol.

[155] Lee SS, Kennedy S, Tolonen AC, Ruvkun G (2003). DAF-16 target genes that control C. elegans life-span and metabolism. Science.

[156] Oh SW, Mukhopadhyay A, Dixit BL, Raha T, Green MR, Tissenbaum HA (2006). Identification of direct DAF-16 targets controlling longevity, metabolism and diapause by chromatin immunoprecipitation. Nat Genet.

[157] Murphy CT, McCarroll SA, Bargmann CI, Fraser A, Kamath RS, Ahringer J, Li H, Kenyon C (2003). Genes that act downstream of DAF-16 to influence the lifespan of Caenorhabditis elegans. Nature.

[158] Schumacher B, van der Pluijm I, Moorhouse MJ, Kosteas T, Robinson AR, Suh Y, Breit TM, van Steeg H, Niedernhofer LJ, van Ijcken W, Bartke A, Spindler SR, Hoeijmakers JH (2008). Delayed and accelerated aging share common longevity assurance mechanisms. PLoS Genet.

[159] Sengupta S, Peterson TR, Sabatini DM (2010). Regulation of the mTOR complex 1 pathway by nutrients, growth factors, and stress. Mol Cell.

[160] Johnson SC, Rabinovitch PS, Kaeberlein M (2013). mTOR is a key modulator of ageing and age-related disease. Nature.

[161] Lamming DW, Ye L, Katajisto P, Goncalves MD, Saitoh M, Stevens DM, Davis JG, Salmon AB, Richardson A, Ahima RS, Guertin DA, Sabatini DM, Baur JA (2012). Rapamycin-induced insulin resistance is mediated by mTORC2 loss and uncoupled from longevity. Science.

[162] Harrison DE, Strong R, Sharp ZD, Nelson JF, Astle CM, Flurkey K, Nadon NL, Wilkinson JE, Frenkel K, Carter CS, Pahor M, Javors MA, Fernandez E, Miller RA (2009). Rapamycin fed late in life extends lifespan in genetically heterogeneous mice. Nature.

[163] Johnson SC, Martin GM, Rabinovitch PS, Kaeberlein M (2013). Preserving youth: does rapamycin deliver?. Sci Transl Med.

[164] Wilkinson JE, Burmeister L, Brooks SV, Chan CC, Friedline S, Harrison DE, Hejtmancik JF, Nadon N, Strong R, Wood LK, Woodward MA, Miller RA (2012). Rapamycin slows aging in mice. Aging Cell.

[165] Mihaylova MM, Shaw RJ (2011). The AMPK signalling pathway coordinates cell growth, autophagy and metabolism. Nat Cell Biol.

[166] Salminen A, Kaarniranta K (2012). AMP-activated protein kinase (AMPK) controls the aging process via an integrated signaling network. Ageing Res Rev.

[167] Cantó C, Auwerx J (2010). AMP-activated protein kinase and its downstream transcriptional pathways. Cell Mol Life Sci.

[168] Reznick RM, Zong H, Li J, Morino K, Moore IK, Yu HJ, Liu ZX, Dong J, Mustard KJ, Hawley SA, Befroy D, Pypaert M, Hardie DG (2007). Aging-associated reductions in AMP-activated protein kinase activity and mitochondrial biogenesis. Cell Metab.

[169] Steinberg GR, Kemp BE (2009). AMPK in health and disease. Physiol Rev.

[170] Houtkooper RH, Cantó C, Wanders RJ, Auwerx J (2010). The secret life of NAD+: an old metabolite controlling new metabolic signaling pathways. Endocr Rev.

[171] Patel M, Shah G (2010). Possible role of hydrogen sulfide in insulin secretion and in development of insulin resistance. J Young Pharm.

[172] Yang W, Yang G, Jia X, Wu L, Wang R (2005). Activation of KATP channels by H2S in rat insulin-secreting cells and the underlying mechanisms. J Physiol.

[173] Łowicka E, Bełtowski J (2007). Hydrogen sulfide (H2S) - the third gas of interest for pharmacologists. Pharmacol Rep.

[174] Liang M, Jin S, Wu DD, Wang MJ, Zhu YC (2015). Hydrogen sulfide improves glucose metabolism and prevents hypertrophy in cardiomyocytes. Nitric Oxide.

[175] Xue R, Hao DD, Sun JP, Li WW, Zhao MM, Li XH, Chen Y, Zhu JH, Ding YJ, Liu J, Zhu YC (2013). Hydrogen sulfide treatment promotes glucose uptake by increasing insulin receptor sensitivity and ameliorates kidney lesions in type 2 diabetes. Antioxid Redox Signal.

[176] Ju Y, Untereiner A, Wu L, Yang G (2015). H2S-induced S-sulfhydration of pyruvate carboxylase contributes to gluconeogenesis in liver cells. Biochim Biophys Acta.

[177] Sun Y, Huang Y, Zhang R, Chen Q, Chen J, Zong Y, Liu J, Feng S, Liu AD, Holmberg L, Liu D, Tang C, Du J, Jin H (2015). Hydrogen sulfide upregulates KATP channel expression in vascular smooth muscle cells of spontaneously hypertensive rats. J Mol Med (Berl).

[178] Ohno K, Okuda K, Uehara T (2015). Endogenous S-sulfhydration of PTEN helps protect against modification by nitric oxide. Biochem Biophys Res Commun.

[179] Greiner R, Pálinkás Z, Bäsell K, Becher D, Antelmann H, Nagy P, Dick TP (2013). Polysulfides link H2S to protein thiol oxidation. Antioxid Redox Signal.

[180] Calenic B, Yaegaki K, Ishkitiev N, Kumazawa Y, Imai T, Tanaka T (2013). p53-Pathway activity and apoptosis in hydrogen sulfide-exposed stem cells separated from human gingival epithelium. J Periodontal Res.

[181] Manna P, Jain SK (2013). L-cysteine and hydrogen sulfide increase PIP3 and AMPK/PPARγ expression and decrease ROS and vascular inflammation markers in high glucose treated human U937 monocytes. J Cell Biochem.

[182] Wei WB, Hu X, Zhuang XD, Liao LZ, Li WD (2014). GYY4137, a novel hydrogen sulfide-releasing molecule, likely protects against high glucose-induced cytotoxicity by activation of the AMPK/mTOR signal pathway in H9c2 cells. Mol Cell Biochem.

[183] Zhou X, Cao Y, Ao G, Hu L, Liu H, Wu J, Wang X, Jin M, Zheng S, Zhen X, Alkayed NJ, Jia J, Cheng J (2014). CaMKKβ-dependent activation of AMP-activated protein kinase is critical to suppressive effects of hydrogen sulfide on neuroinflammation. Antioxid Redox Signal.

[184] Kundu S, Pushpakumar S, Khundmiri SJ, Sen U (2014). Hydrogen sulfide mitigates hyperglycemic remodeling via liver kinase B1-adenosine mono-phosphate-activated protein kinase signaling. Biochim Biophys Acta.

[185] Yang M, Huang Y, Chen J, Chen YL, Ma JJ, Shi PH (2014). Activation of AMPK participates hydrogen sulfide-induced cyto-protective effect against dexa-methasone in osteoblastic MC3T3-E1 cells. Biochem Biophys Res Commun.

[186] Xie H, Xu Q, Jia J, Ao G, Sun Y, Hu L, Alkayed NJ, Wang C, Cheng J (2015). Hydrogen sulfide protects against myocardial ischemia and reperfusion injury by activating AMP-activated protein kinase to restore autophagic flux. Biochem Biophys Res Commun.

[187] Wiliński B, Wiliński J, Somogyi E, Piotrowska J, Opoka W (2013). Metformin raises hydrogen sulfide tissue concentrations in various mouse organs. Pharmacol Rep.

[188] Lee HJ, Mariappan MM, Feliers D, Cavaglieri RC, Sataranatarajan K, Abboud HE, Choudhury GG, Kasinath BS (2012). Hydrogen sulfide inhibits high glucose-induced matrix protein synthesis by activating AMP-activated protein kinase in renal epithelial cells. J Biol Chem.

[189] Wu YC, Wang XJ, Yu L, Chan FK, Cheng AS, Yu J, Sung JJ, Wu WK, Cho CH (2012). Hydrogen sulfide lowers proliferation and induces protective autophagy in colon epithelial cells. PLoS One.

[190] Zhou Y, Wang D, Gao X, Lew K, Richards AM, Wang P (2014). mTORC2 phosphorylation of Akt1: a possible mechanism for hydrogen sulfide-induced cardioprotection. PLoS One.

[191] Cheng P, Chen K, Xia Y, Dai W, Wang F, Shen M, Wang C, Yang J, Zhu R, Zhang H, Li J, Zheng Y, Wang J (2014). Hydrogen sulfide, a potential novel drug, attenuates concanavalin A-induced hepatitis. Drug Des Devel Ther.

[192] Xie L, Feng H, Li S, Meng G, Liu S, Tang X, Ma Y, Han Y, Xiao Y, Gu Y, Shao Y, Park CM, Xian M (2015). SIRT3 mediates the anti-oxidant effect of hydrogen sulfide in endothelial cells. Antioxid Redox Signal.

[193] Hu Y, Li R, Yang H, Luo H, Chen Z (2015). Sirtuin 6 is essential for sodium sulfide-mediated cytoprotective effect in ischemia/reperfusion-stimulated brain endothelial cells. J Stroke Cerebrovasc Dis.

[194] Kroemer G, Galluzzi L, Brenner C (2007). Mitochondrial membrane permeabilization in cell death. Physiol Rev.

[195] Raffaello A, Rizzuto R (2011). Mitochondrial longevity pathways. Biochim Biophys Acta.

[196] Green DR, Galluzzi L, Kroemer G (2011). Mitochondria and the autophagy-inflammation-cell death axis in organismal aging. Science.

[197] Cui H, Kong Y, Zhang H (2012). Oxidative stress, mitochondrial dysfunction, and aging. J Signal Transduct.

[198] Sena LA, Chandel NS (2012). Physiological roles of mitochondrial reactive oxygen species. Mol Cell.

[199] Hawley SA, Ross FA, Chevtzoff C, Green KA, Evans A, Fogarty S, Towler MC, Brown LJ, Ogunbayo OA, Evans AM, Hardie DG (2010). Use of cells expressing gamma subunit variants to identify diverse mechanisms of AMPK activation. Cell Metab.

[200] Onken B, Driscoll M (2010). Metformin induces a dietary restriction-like state and the oxidative stress response to extend C. elegans Healthspan via AMPK, LKB1, and SKN-1. PLoS One.

[201] Harman D (1972). The biologic clock: the mitochondria?. J Am Geriatr Soc.

[202] Chomyn A, Attardi G (2003). MtDNA mutations in aging and apoptosis. Biochem Biophys Res Commun.

[203] Hiona A, Leeuwenburgh C (2008). The role of mitochondrial DNA mutations in aging and sarcopenia: implications for the mitochondrial vicious cycle theory of aging. Exp Gerontol.

[204] Perez VI, Bokov A, Van Remmen H, Mele J, Ran Q, Ikeno Y, Richardson A (2009). Is the oxidative stress theory of aging dead?. Biochim Biophys Acta.

[205] Ristow M, Schmeisser S (2011). Extending life span by increasing oxidative stress. Free Radic Biol Med.

[206] Sies H (1993). Strategies of antioxidant defense. Eur J Biochem.

[207] Dröge W (2002). Free radicals in the physiological control of cell function. Physiol Rev.

[208] Haigis MC, Yankner BA (2010). The aging stress response. Mol Cell.

[209] Yun J, Finkel T (2014). Mitohormesis. Cell Metab.

[210] Calabrese EJ, Baldwin LA (2002). Defining hormesis. Hum Exp Toxicol.

[211] Calabrese V, Cornelius C, Cuzzocrea S, Iavicoli I, Rizzarelli E, Calabrese EJ (2011). Hormesis, cellular stress response and vitagenes as critical determinants in aging and longevity. Mol Aspects Med.

[212] Hekimi S, Lapointe J, Wen Y (2011). Taking a “good” look at free radicals in the aging process. Trends Cell Biol.

[213] Kimura Y, Goto Y, Kimura H (2010). Hydrogen sulfide increases glutathione production and suppresses oxidative stress in mitochondria. Antioxid Redox Signal.

[214] Kimura Y, Kimura H (2004). Hydrogen sulfide protects neurons from oxidative stress. FASEB J.

[215] Bos EM, Wang R, Snijder PM, Boersema M, Damman J, Fu M, Moser J, Hillebrands JL, Ploeg RJ, Yang G, Leuvenink HG, van Goor H (2013). Cystathionine γ-lyase protects against renal ischemia/reperfusion by modulating oxidative stress. J Am Soc Nephrol.

[216] Sun WH, Liu F, Chen Y, Zhu YC (2012). Hydrogen sulfide decreases the levels of ROS by inhibiting mitochondrial complex IV and increasing SOD activities in cardiomyocytes under ischemia/reperfu-sion. Biochem Biophys Res Commun.

[217] Calvert JW, Jha S, Gundewar S, Elrod JW, Ramachandran A, Pattillo CB, Kevil CG, Lefer DJ (2009). Hydrogen sulfide mediates cardioprotection through Nrf2 signaling. Circ Res.

[218] Yang G, Zhao K, Ju Y, Mani S, Cao Q, Puukila S, Khaper N, Wu L, Wang R (2013). Hydrogen sulfide protects against cellular senescence via S-sulfhydration of Keap1 and activation of Nrf2. Antioxid Redox Signal.

[219] Goubern M, Andriamihaja M, Nübel T, Blachier F, Bouillaud F (2007). Sulfide, the first inorganic substrate for human cells. FASEB J.

[220] Hildebrandt TM, Grieshaber MK (2008). Three enzymatic activities catalyze the oxidation of sulfide to thiosulfate in mammalian and invertebrate mitochondria. FEBS J.

[221] Lagoutte E, Mimoun S, Andriamihaja M, Chaumontet C, Blachier F, Bouillaud F (2010). Oxidation of hydrogen sulfide remains a priority in mammalian cells and causes reverse electron transfer in colonocytes. Biochim Biophys Acta.

[222] Guo W, Kan JT, Cheng ZY, Chen JF, Shen YQ, Xu J, Wu D, Zhu YZ (2012). Hydrogen sulfide as an endogenous modulator in mitochondria and mitochondria dysfunction. Oxid Med Cell Longev.

[223] Bos EM, Leuvenink HG, Snijder PM, Kloosterhuis NJ, Hillebrands JL, Leemans JC, Florquin S, van Goor H (2009). Hydrogen sulfide-induced hypometabolism prevents renal ischemia/reperfusion injury. J Am Soc Nephrol.

[224] Bos EM, Snijder PM, Jekel H, Weij M, Leemans JC, van Dijk MC, Hillebrands JL, Lisman T, van Goor H, Leuvenink HG (2012). Beneficial effects of gaseous hydrogen sulfide in hepatic ischemia/reperfusion injury. Transpl Int.

[225] Hayflick L, Moorhead PS (1961). The serial cultivation of human diploid cell strains. Exp Cell Res.

[226] van Deursen JM (2014). The role of senescent cells in ageing. Nature.

[227] Kuilman T, Michaloglou C, Mooi WJ, Peeper DS (2010). The essence of senescence. Genes Dev.

[228] Matheu A, Maraver A, Klatt P, Flores I, Garcia-Cao I, Borras C, Flores JM, Viña J, Blasco MA, Serrano M (2007). Delayed ageing through damage protection by the Arf/p53 pathway. Nature.

[229] Matheu A, Maraver A, Collado M, Garcia-Cao I, Cañamero M, Borras C, Flores JM, Klatt P, Viña J, Serrano M (2009). Anti-aging activity of the Ink4/Arf locus. Aging Cell.

[230] Cao L, Li W, Kim S, Brodie SG, Deng CX (2003). Senescence, aging, and malignant transformation mediated by p53 in mice lacking the Brca1 full-length isoform. Genes Dev.

[231] Baker DJ, Wijshake T, Tchkonia T, LeBrasseur NK, Childs BG, van de Sluis B, Kirkland JL, van Deursen JM (2011). Clearance of p16Ink4a-positive senescent cells delays ageing-associated disorders. Nature.

[232] Bodnar AG, Ouellette M, Frolkis M, Holt SE, Chiu CP, Morin GB, Harley CB, Shay JW, Lichtsteiner S, Wright WE (1998). Extension of life-span by introduction of telomerase into normal human cells. Science.

[233] Sedelnikova OA, Horikawa I, Zimonjic DB, Popescu NC, Bonner WM, Barrett JC (2004). Senescing human cells and ageing mice accumulate DNA lesions with unrepairable double-strand breaks. Nat Cell Biol.

[234] von Zglinicki T (2002). Oxidative stress shortens telomeres. Trends Biochem Sci.

[235] Aird KM, Zhang G, Li H, Tu Z, Bitler BG, Garipov A, Wu H, Wei Z, Wagner SN, Herlyn M, Zhang R (2013). Suppression of nucleotide metabolism underlies the establishment and maintenance of oncogene-induced senescence. Cell Reports.

[236] Di Micco R, Fumagalli M, Cicalese A, Piccinin S, Gasparini P, Luise C, Schurra C, Garre' M, Nuciforo PG, Bensimon A, Maestro R, Pelicci PG, d'Adda di Fagagna F (2006). Oncogene-induced senescence is a DNA damage response triggered by DNA hyper-replication. Nature.

[237] Serrano M, Lin AW, McCurrach ME, Beach D, Lowe SW (1997). Oncogenic ras provokes premature cell senescence associated with accumulation of p53 and p16INK4a. Cell.

[238] Jeck WR, Siebold AP, Sharpless NE (2012). Review: a meta-analysis of GWAS and age-associated diseases. Aging Cell.

[239] Krishnamurthy J, Torrice C, Ramsey MR, Kovalev GI, Al-Regaiey K, Su L, Sharpless NE (2004). Ink4a/Arf expression is a biomarker of aging. J Clin Invest.

[240] Ressler S, Bartkova J, Niederegger H, Bartek J, Scharffetter-Kochanek K, Jansen-Dürr P, Wlaschek M (2006). p16INK4A is a robust in vivo biomarker of cellular aging in human skin. Aging Cell.

[241] Kaplon J, Zheng L, Meissl K, Chaneton B, Selivanov VA, Mackay G, van der Burg SH, Verdegaal EM, Cascante M, Shlomi T, Gottlieb E, Peeper DS (2013). A key role for mitochondrial gatekeeper pyruvate dehydro-genase in oncogene-induced senescence. Nature.

[242] Kondoh H, Lleonart ME, Gil J, Wang J, Degan P, Peters G, Martinez D, Carnero A, Beach D (2005). Glycolytic enzymes can modulate cellular life span. Cancer Res.

[243] Dörr JR, Yu Y, Milanovic M, Beuster G, Zasada C, Däbritz JH, Lisec J, Lenze D, Gerhardt A, Schleicher K, Kratzat S, Purfürst B, Walenta S (2013). Synthetic lethal metabolic targeting of cellular senescence in cancer therapy. Nature.

[244] Rodier F, Campisi J (2011). Four faces of cellular senescence. J Cell Biol.

[245] Wang C, Jurk D, Maddick M, Nelson G, Martin-Ruiz C, von Zglinicki T (2009). DNA damage response and cellular senescence in tissues of aging mice. Aging Cell.

[246] Baker DJ, Perez-Terzic C, Jin F, Pitel KS, Niederländer NJ, Jeganathan K, Yamada S, Reyes S, Rowe L, Hiddinga HJ, Eberhardt NL, Terzic A, van Deursen JM (2008). Opposing roles for p16Ink4a and p19Arf in senescence and ageing caused by BubR1 insufficiency. Nat Cell Biol.

[247] Xie ZZ, Shi MM, Xie L, Wu ZY, Li G, Hua F, Bian JS (2014). Sulfhydration of p66Shc at cysteine59 mediates the antioxidant effect of hydrogen sulfide. Antioxid Redox Signal.

[248] Predmore BL, Alendy MJ, Ahmed KI, Leeuwenburgh C, Julian D (2010). The hydrogen sulfide signaling system: changes during aging and the benefits of caloric restriction. Age (Dordr).

[249] Liang R, Ghaffari S (2014). Stem cells, redox signaling, and stem cell aging. Antioxid Redox Signal.

[250] He S, Nakada D, Morrison SJ (2009). Mechanisms of stem cell self-renewal. Annu Rev Cell Dev Biol.

[251] Rossi DJ, Bryder D, Seita J, Nussenzweig A, Hoeijmakers J, Weissman IL (2007). Deficiencies in DNA damage repair limit the function of haematopoietic stem cells with age. Nature.

[252] Flores I, Cayuela ML, Blasco MA (2005). Effects of telomerase and telomere length on epidermal stem cell behavior. Science.

[253] Sharpless NE, DePinho RA (2007). How stem cells age and why this makes us grow old. Nat Rev Mol Cell Biol.

[254] Flores I, Benetti R, Blasco MA (2006). Telomerase regulation and stem cell behaviour. Curr Opin Cell Biol.

[255] Hiyama E, Hiyama K (2007). Telomere and telomerase in stem cells. Br J Cancer.

[256] Kastan MB, Bartek J (2004). Cell-cycle checkpoints and cancer. Nature.

[257] Ruzankina Y, Brown EJ (2007). Relationships between stem cell exhaustion, tumour suppression and ageing. Br J Cancer.

[258] Cerletti M, Jang YC, Finley LW, Haigis MC, Wagers AJ (2012). Short-term calorie restriction enhances skeletal muscle stem cell function. Cell Stem Cell.

[259] Yilmaz OH, Katajisto P, Lamming DW, Gültekin Y, Bauer-Rowe KE, Sengupta S, Birsoy K, Dursun A, Yilmaz VO, Selig M, Nielsen GP, Mino-Kenudson M, Zukerberg LR (2012). mTORC1 in the Paneth cell niche couples intestinal stem-cell function to calorie intake. Nature.

[260] Castilho RM, Squarize CH, Chodosh LA, Williams BO, Gutkind JS (2009). mTOR mediates Wnt-induced epidermal stem cell exhaustion and aging. Cell Stem Cell.

[261] Chen C, Liu Y, Liu Y, Zheng P (2009). mTOR regulation and therapeutic rejuvenation of aging hematopoietic stem cells. Sci Signal.

[262] Chambers SM, Shaw CA, Gatza C, Fisk CJ, Donehower LA, Goodell MA (2007). Aging hematopoietic stem cells decline in function and exhibit epigenetic dysregulation. PLoS Biol.

[263] Su Y, Liu D, Liu Y, Zhang C, Wang J, Wang S (2015). Physiological level of endogenous hydrogen sulfide maintains the proliferation and differentiation capacity of periodontal ligament stem cells. J Periodontol.

[264] Wang Z, Liu DX, Wang FW, Zhang Q, Du ZX, Zhan JM, Yuan QH, Ling EA, Hao AJ (2013). L-Cysteine promotes the proliferation and differentiation of neural stem cells via the CBS/H2S pathway. Neuroscience.

[265] Liu D, Wang Z, Zhan J, Zhang Q, Wang J, Zhang Q, Xian X, Luan Q, Hao A (2014). Hydrogen sulfide promotes proliferation and neuronal differentiation of neural stem cells and protects hypoxia-induced decrease in hippocampal neurogenesis. Pharmacol Biochem Behav.

[266] Dongó E, Benkő Z, Csizmazia Á, Marosi G, Grottke A, Jücker M, Schumacher U, Kiss L (2014). H2S preconditioning of human adipose tissue-derived stem cells increases their efficacy in an in vitro model of cell therapy for simulated ischemia. Life Sci.

[267] Liu Y, Yang R, Liu X, Zhou Y, Qu C, Kikuiri T, Wang S, Zandi E, Du J, Ambudkar IS, Shi S (2014). Hydrogen sulfide maintains mesenchymal stem cell function and bone homeostasis via regulation of Ca(2+) channel sulfhydration. Cell Stem Cell.

[268] Li C, Guo Z, Guo B, Xie Y, Yang J, Wang A (2014). Inhibition of the endogenous CSE/H2S system contributes to hypoxia and serum deprivation-induced apoptosis in mesenchymal stem cells. Mol Med Rep.

[269] Guo Z, Li CS, Wang CM, Xie YJ, Wang AL (2015). CSE/H2S system protects mesenchymal stem cells from hypoxia and serum deprivation induced apoptosis via mitochondrial injury, endoplasmic reticulum stress and PI3K/Akt activation pathways. Mol Med Rep.

[270] Ostrakhovitch EA, Akakura S, Sanokawa-Akakura R, Goodwin S, Tabibzadeh S (2015). Dedifferentiation of cancer cells following recovery from a potentially lethal damage is mediated by H2S-Nampt. Exp Cell Res.

[271] Nelson G, Wordsworth J, Wang C, Jurk D, Lawless C, Martin-Ruiz C, von Zglinicki T (2012). A senescent cell bystander effect: senescence-induced senescence. Aging Cell.

[272] Zhang G, Li J, Purkayastha S, Tang Y, Zhang H, Yin Y, Li B, Liu G, Cai D (2013). Hypothalamic programming of systemic ageing involving IKK-β, NF-κB and GnRH. Nature.

[273] Laplante M, Sabatini DM (2012). mTOR signaling in growth control and disease. Cell.

[274] Rando TA, Chang HY (2012). Aging, rejuvenation, and epigenetic reprogramming: resetting the aging clock. Cell.

[275] Salminen A, Kaarniranta K, Kauppinen A (2012). Inflammaging: disturbed interplay between autophagy and inflammasomes. Aging (Albany NY).

[276] Tabas I (2010). Macrophage death and defective inflammation resolution in atherosclerosis. Nat Rev Immunol.

[277] Deeks SG (2011). HIV infection, inflammation, immuno-senescence, and aging. Annu Rev Med.

[278] Xie J, Zhang X, Zhang L (2013). Negative regulation of inflammation by SIRT1. Pharmacol Res.

[279] Durieux J, Wolff S, Dillin A (2011). The cell-non-autonomous nature of electron transport chain-mediated longevity. Cell.

[280] Lavasani M, Robinson AR, Lu A, Song M, Feduska JM, Ahani B, Tilstra JS, Feldman CH, Robbins PD, Niedernhofer LJ, Huard J (2012). Muscle-derived stem/progenitor cell dysfunction limits healthspan and lifespan in a murine progeria model. Nat Commun.

[281] Tomás-Loba A, Flores I, Fernández-Marcos PJ, Cayuela ML, Maraver A, Tejera A, Borrás C, Matheu A, Klatt P, Flores JM, Viña J, Serrano M, Blasco MA (2008). Telomerase reverse transcriptase delays aging in cancer-resistant mice. Cell.

[282] Freije JM, López-Otín C (2012). Reprogramming aging and progeria. Curr Opin Cell Biol.

[283] Claesson MJ, Jeffery IB, Conde S, Power SE, O'Connor EM, Cusack S, Harris HM, Coakley M, Lakshminarayanan B, O'Sullivan O, Fitzgerald GF, Deane J, O'Connor M (2012). Gut microbiota composition correlates with diet and health in the elderly. Nature.

[284] Ottaviani E, Ventura N, Mandrioli M, Candela M, Franchini A, Franceschi C (2011). Gut microbiota as a candidate for lifespan extension: an ecological/evolu-tionnary perspective targeted on living organisms as metaorganisms. Biogerontology.

[285] Yang G (2011). Hydrogen sulfide in cell survival: a double-edged sword. Expert Rev Clin Pharmacol.

[286] Fox B, Schantz JT, Haigh R, Wood ME, Moore PK, Viner N, Spencer JP, Winyard PG, Whiteman M (2012). Inducible hydrogen sulfide synthesis in chondrocytes and mesenchymal progenitor cells: is H2S a novel cytoprotective mediator in the inflamed joint?. J Cell Mol Med.

[287] Xu W, Chen J, Lin J, Liu D, Mo L, Pan W, Feng J, Wu W, Zheng D (2015). Exogenous H2S protects H9c2 cardiac cells against high glucose-induced injury and inflammation by inhibiting the activation of the NF-κB and IL-1β pathways. Int J Mol Med.

[288] Shimada S, Fukai M, Wakayama K, Ishikawa T, Kobayashi N, Kimura T, Yamashita K, Kamiyama T, Shimamura T, Taketomi A, Todo S (2015). Hydrogen sulfide augments survival signals in warm ischemia and reperfusion of the mouse liver. Surg Today.

[289] Gao L, Cheng C, Sparatore A, Zhang H, Wang C (2015). Hydrogen sulfide inhibits human platelet aggregation in vitro in part by interfering gap junction channels: effects of ACS14, a hydrogen sulfide-releasing aspirin. Heart Lung Circ.

[290] Munaron L, Avanzato D, Moccia F, Mancardi D (2013). Hydrogen sulfide as a regulator of calcium channels. Cell Calcium.

[291] Morikawa T, Kajimura M, Nakamura T, Hishiki T, Nakanishi T, Yukutake Y, Nagahata Y, Ishikawa M, Hattori K, Takenouchi T, Takahashi T, Ishii I, Matsubara K (2012). Hypoxic regulation of the cerebral microcirculation is mediated by a carbon monoxide-sensitive hydrogen sulfide pathway. Proc Natl Acad Sci USA.

